# The TRPM7 channel reprograms cellular glycolysis to drive tumorigenesis and angiogenesis

**DOI:** 10.1038/s41419-023-05701-7

**Published:** 2023-03-06

**Authors:** Wanzhou Wu, Xuan Wang, Longsheng Liao, Jing Chen, Yue Wang, Meilian Yao, Lingping Zhu, Jiayu Li, Xuan Wang, Alex F. Chen, Guogang Zhang, Zheng Zhang, Yongping Bai

**Affiliations:** 1 Department of Cardiology, The Third Xiangya Hospital, Central South University, Changsha, China; 2 Center for Vascular Disease and Translational Medicine, Department of Cardiology, The Third Xiangya Hospital, Central South University, Changsha, China; 3 Department of Geriatric Medicine, Xiangya Hospital, Central South University, Changsha, China; 4 College of Pharmacy, Hunan University of Chinese Medicine, Changsha, China; 5 Department of Pharmacology, Hunan Key Laboratory of Cardiovascular Research, Xiangya School of Pharmaceutical Sciences, Central South University, Changsha, China; 6 Institute of Development and Regenerative Medicine, Xinhua Hospital Affiliated to Shanghai Jiaotong University School of Medicine, Shanghai, China; 7 National Clinical Research Center for Geriatric Disorders, Xiangya Hospital, Central South University, Changsha, China

**Keywords:** Ion channel signalling, Cancer metabolism

## Abstract

Cancer or endothelial cells preferably catabolize glucose through aerobic glycolysis rather than oxidative phosphorylation. Intracellular ionic signaling has been shown to regulate glucose metabolism, but the underlying ion channel has yet to be identified. RNA-seq, metabolomics and genetic assay revealed that the TRPM7 channel regulated cellular glycolysis. Deletion of TRPM7 suppressed cancer cell glycolysis and reduced the xenograft tumor burden. Deficiency of endothelial TRPM7 inhibited postnatal retinal angiogenesis in mice. Mechanistically, TRPM7 transcriptionally regulated the solute carrier family 2 member 3 (SLC2A3, also known as GLUT3) via Ca^2+^ influx-induced calcineurin activation. Furthermore, CREB-regulated transcription coactivator 2 (CRTC2) and CREB act downstream of calcineurin to relay Ca^2+^ signal to SLC2A3 transcription. Expression of the constitutively active CRTC2 or CREB in TRPM7 knockout cell normalized glycolytic metabolism and cell growth. The TRPM7 channel represents a novel regulator of glycolytic reprogramming. Inhibition of the TRPM7-dependent glycolysis could be harnessed for cancer therapy.

## Introduction

Most cells consume glucose for energy production and biomass buildup. Under aerobic conditions, glucose is preferably catabolized through oxidative phosphorylation in the mitochondria. Oxidative phosphorylation via the tricarboxylic acid cycle (TCA) is believed to be more efficient in yielding ATP, and is the major route of glucose metabolism in a vast majority of cells. However, some hyperactive cells, such as cancer and proliferative endothelial cells, utilize glucose via aerobic glycolysis, given that glycolysis requires less rate-limiting enzymatic steps and generates ATP in an accelerated manner [1]. Some cancer cells are addicted to glycolysis, generating lactate to remodel the acidic microenvironment for tumor growth, metastasis and pathological angiogenesis [2]. Like cancer cells, the healthy vascular endothelial cells (VECs) have recently been demonstrated to prefer glycolysis even if oxygen is sufficient in the oxygenated blood [1, 3]. Moreover, VECs metabolize glucose through glycolysis to drive angiogenesis [1, 3]. In light of the pathogenic role of glycolytic reprogramming, inhibition of glycolysis has been pursued as a therapeutic strategy for suppressing oncogenesis and pathological angiogenesis [2, 4].

Cells are endowed with a multitude of ion channels that finely tune the intracellular signaling, including cellular glycolysis [5]. Intracellular ionic perturbation has been implicated in glycolytic reprogramming, of which calcium signaling has been best studied. Store-operated calcium entry reprograms glycolysis via glucose transporter GLUT3 enabling T cell expansion [6]. Beige fat enhances glycolysis via SERCA2b-mediated calcium cycling [7]. Intracellular calcium signaling is tightly regulated through a complex cascade of signaling molecules, including membrane Ca^2+^ channels, and calcium-sensitive protein known as calcium effectors [8]. Calcium channels are diverse in its identity, modulation, signal transduction and cellular functions. Given the complexity of calcium signaling, further exploration of calcium signaling in metabolic reprogramming would yield novel insights into the pathogenesis of diseases such as cancer. Here, through an integrated set of approaches, we identified the transient receptor potential melastatin 7 (TRPM7), a member of the TRP channel superfamily, as a gatekeeper of cellular glycolysis in glycolytic cancer and endothelial cells. We also demonstrated that the TRPM7 channel is required for oncogenesis and angiogenesis.

TRPM7 is a chanzyme containing both an ion channel domain and a carboxyl-terminal kinase domain [9, 10]. It acts as a channel conduit for Ca^2+^, Mg^2+^, as well as a kinase that epigenetically regulates gene transcription [11]. TRPM7 has been implicated in embryogenesis, cardiogenesis, vascular diseases [12–15], but whether this multifunctional protein pathogenically controls cellular glycolysis remains unknown. The present study reveals that TRPM7 channel transcriptionally regulates the glucose transporter GLUT3 via its calcium channel functionality. Ca^2+^ influx through the TRPM7 channel activates calcineurin, which in turn dephosphorylates CREB-regulated transcription coactivator 2 (CRTC2) followed by CRTC2 nuclear translocation and activation of cAMP-response element binding protein (CREB). Hence, our data suggest that block of TRPM7 channel-dependent cellular glycolysis is a novel therapeutic strategy for cancer therapy and pathological angiogenesis.

## Methods

A concise description of the methodology is provided below. For detailed information, please refer to the online supplementary materials.

### *Trpm7* knockout (KO) mice

*Trpm7*^*fl/fl*^ mice were purchased from the Jackson Laboratory. To generate endothelium-selective deficiency of *Trpm7 (Trpm7*^*ECKO*^*)*, *Trpm7*^*fl/fl*^ mice were crossed with *Tie2-Cre* mice. *Cre*-positive male mice were used for intercrossing to prevent recombination in the female germline. Animal experiments were performed in accordance with guidelines and protocols approved by the Ethics Committee of Xiangya Hospital (No. 2022020390).

### Retinal vessel growth analysis

As previously reported [3], after euthanasia, whole eyes from mice on postnatal day (P) 6.5 were fixed in 4% paraformaldehyde for 1 h at room temperature (RT). After blocking and permeabilization in retina-blocking buffer (1% bovine serum albumin [BSA] and 0.5% Triton X-100 in PBS) overnight, the retinas were incubated at 4 °C in PBLEC buffer (1 mM CaCl_2_, 1 mM MgCl_2_, 0.1 mM MnCl_2_, and 1% Triton X-100 in PBS) with Alexa Fluor 488-conjugated IB4 (Thermo Fisher, I21411, 1:200) overnight. After washing with PBLEC buffer, the retinas were dissected into four leaflets. The retinas were examined by confocal laser microscopy (Zeiss LSM 900 with Airyscan2).

### Subcutaneous tumor xenograft model

BALB/c-nu mice were purchased from Hunan SJA Laboratory Animal Co., Ltd. (Changsha, China). TRPM7 knockout T24 or WT cells (5 × 10^6^) were diluted in 200 µL of PBS and Matrigel. Tumors were evaluated and recorded regularly after cell transplantation. The volume of the tumors was measured as follows: V = D × d^2^ × π/6, where V is volume, D is the longest diameter of the bulk tumor, and d is the shortest diameter of the bulk tumor. No mice died during the experiment.

### Cell culture

Primary HUVECs were gained from ScienCell and cultured in ECM (ScienCell, 1001) supplemented with EC growth supplement (ECGS), 5% fetal bovine serum (FBS), and a penicillin/streptomycin cocktail. HUVECs were used for analysis before the 5th passage. mLECs were isolated as described. In brief, the lungs of adult mice were harvested and incubated with dispase (Roche, 10269638001). The homogenate was filtered through 100-μm and 40-μm cell strainers. The cell suspension was collected and incubated with mouse CD31 microbeads (Miltenyi Biotec, 130-097-418). The beads were washed with Dulbecco’s PBS (DPBS) supplemented with 1% FBS and then used for total RNA isolation. HEK293T cells were gained from the National Collection of Authenticated Cell Cultures and cultured in DMEM supplemented with 10% FBS. Wild type and TRPM7 KO T24 human urinary bladder carcinoma cells were generated in Dr. Zheng Zhang’s lab. The cells were authenticated through short tandem repeat (STR) by Genetic Testing Biotechnology Corporation (Suzhou, China) and cultured in DMEM supplemented with 10% FBS.

### Transcriptomics

The bulk RNA sequencing was performed by Metware Co., LTD. The data was analyzed by gene set enrichment analysis (GSEA) from Molecular signatures Database (MsugDB) v.4.0. The GESA was performed with WT cells being controls for TRPM7KO cells. The data that supports the findings of this study are available in the supplementary material of this article.

### Cell growth assays

The proliferation of cancer cells and HUVECs was measured by Cell Count Kit 8 (Dojindo) and EdU staining (RiboBio). The migration was assay by wound-healing assay. Endothelial vessel formation ability was measured by tube-formation.

### Metabolic tests

The glycolysis and oxidative phosphorylation were measured by Seahorse (Agilent). The lactate production was tested by Lactate Assay Kit (Abcam). Metabolic flux assay was performed by Suzhou PANOMIX Biomedical Tech Co., LTD.

### Statistical analysis

Statistical analysis was performed with GraphPad Prism software (v. 9.2.0). All experimental values are presented as the mean ± s.e.m. Statistical significance was assessed by ANOVA or Student’s *t* test. A *P* value < 0.05 was considered to be statistically significant. All studies were repeated by two independent experimenters at least three times. No randomization or blinding was enforced, and no animals were eliminated from the analyses.

## Results

### TRPM7 knockout reduces glucose uptake and catabolism

To search for the ion channel candidate linking ionic signaling to cellular glycolysis, we set out to screen the expression of the TRP channel superfamily (27 mammalian members in total) in T24 and MCF7 cells, cancer cell line with active glycolysis [1, 16]. TRPM7 stood out as an intriguing hit, based on: (1) its highest expression among TRP channels (Fig. S1A, B); (2) its multiple functions as Ca^2+^/Mg^2+^ channels and α kinase, which may directly regulate glycolysis via cytosolic Mg^2+^ or indirectly via Ca^2+^/kinase-mediated transcriptional programs.

**Fig. 1:**
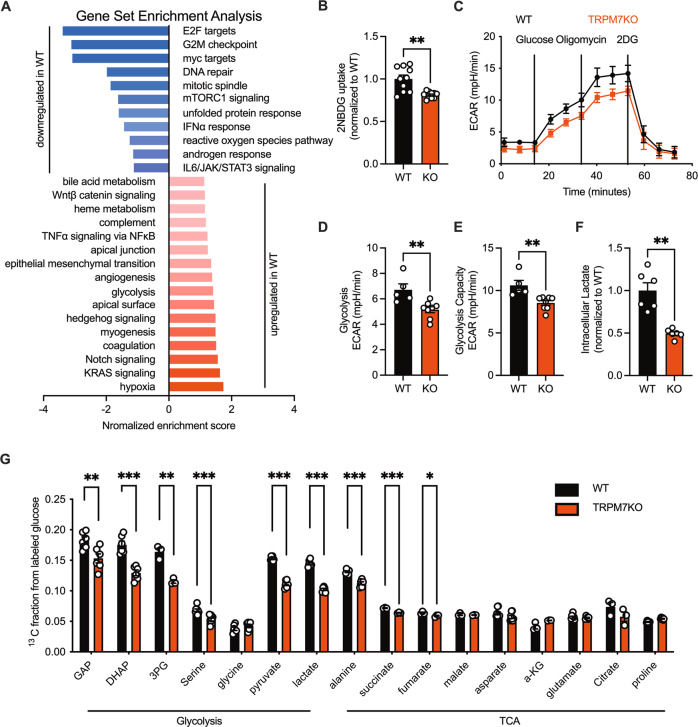
TRPM7 knockout impairs glucose catabolism. **A** Gene set enrichment analysis (GSEA) of Hallmark gene sets in TRPM7KO versus WT T24 cells. **B** Glucose uptake analysis by 2-deoxy-2-[(7-nitro-2,1,3-benzoxadiazol-4-yl)amino]-D-glucose (2NBDG). **C**–**E** Glycolysis was measured by Extracellular acidification rates (ECAR) in WT and TRPM7KO cells under basal conditions and in response to 10 mM glucose, 3 μM oligomycin or 100 mM 2-deoxy-D-glucose (2-DG). **F** Relative intracellular lactate in WT or TRPM7KO cells. **G** Glycolytic intermediates and metabolites quantified by mass spectrometry. Two groups of samples were compared by unpaired two-tailed Student’s *t*-test.

TRPM7 has been conventionally genetically manipulated for in vitro functional analysis, in most cases gene knockdown using small interfering RNA (siRNA). Gene knockout is believed to be more reproducible, prompting us to make a TRPM7 knockout (hereafter referred to as TRPM7KO) human urinary bladder carcinoma cell line (T24 cell) using the CRISPR/Cas9 technology (Fig. S2A–C). RNA-seq transcriptomic analysis was performed in TRPM7KO cells to examine the impact of TRPM7 loss on gene transcription (Fig. S3A). Gene set enrichment analysis (GSEA) revealed that TRPM7 KO downregulated glycolysis and angiogenesis-related genes (Fig. 1A). As revealed by Kyoto encyclopedia of genes & genomes (KEGG) analysis, cell survival & growth-related cell cycle & DNA replication signaling was enriched in TRPM7-intact cells (Fig. S3B). To further functionally characterize the phenotype of TRPM7 knockout, we checked the glucose uptake, since transporter-mediated transmembrane glucose transport is the initiating step of glucose catabolism, and its efficiency limits glucose utilization. Analysis of glucose uptake using a fluorescent glucose analog showed that TRPM7KO cells exhibited reduced glucose uptake (Fig. 1B). Next, we examined glycolysis as well as oxidative phosphorylation, the 2 major ATP-generating means in cancer cells. As extracellular acidification rate (ECAR) and oxygen consumption rate (OCR) revealed, TRPM7 knockout markedly suppressed glycolysis, in addition to oxidative phosphorylation and ATP production (Fig. 1C–E, S4A–E). Also, TRPM7KO caused less lactate production (Fig. 1F). The results were further strengthened by isotope labeled glucose utilization analysis, which showed defective glycolysis and TCA in TRPM7KO cells (Fig. 1G).

Cancer cells enforce glycolysis for tumor growth, and this serves as the rationale for pursing glucose catabolism block as an anti-cancer maneuver. Of significance, TRPM7 KO considerably inhibited cancer cell proliferation, invasion and migration (Fig. S5A–E). To corroborate the in vitro results, we developed a xenograft tumor model using TRPM7KO cells; smaller tumor volume was observed in TRPM7KO tumor-burdened mice (Fig. S5F–G). Together, our data suggest that TRPM7 controls glucose uptake and utilization to boost tumor growth.

### The TRPM7- SLC2A3 axis controls glycolytic metabolism and cell growth

**Fig. 2:**
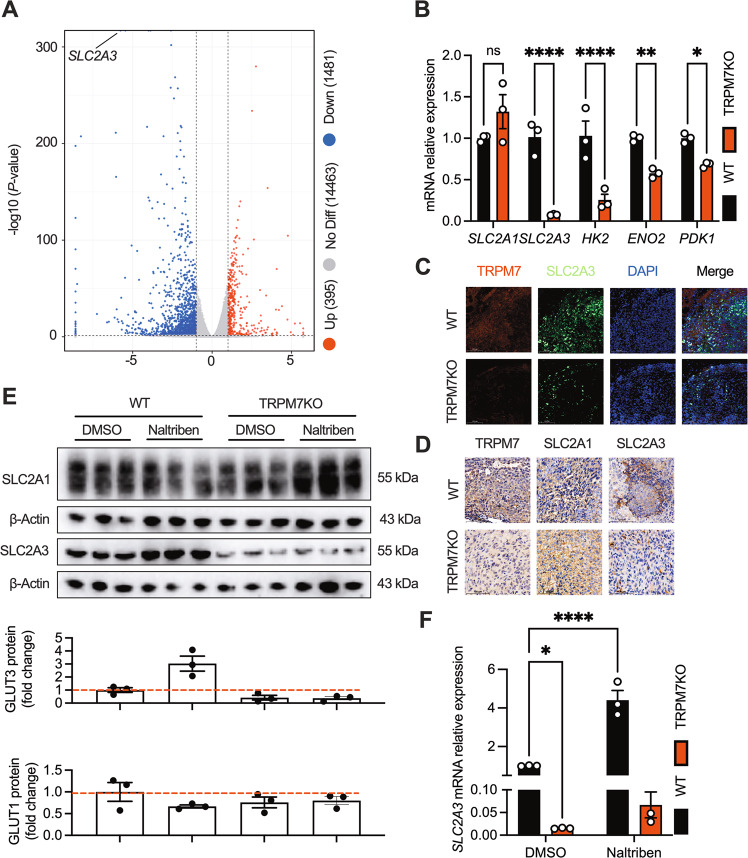
TRPM7 deletion diminishes SLC2A3 expression. **A** Volcano plots of glucose metabolism-related genes that are induced in WT versus TRPM7KO cells. Genes with a fold-change ≥2 and *P* value of < 0.05 are shown. **B** RT-qPCR analysis validating the decreased expression of glucose catabolic genes from RNA-seq in TRPM7KO cells. **C** Immunofluorescent staining of TRPM7, SLC2A3 and DAPI in the xenograft tumor (scale bars, 100 μm). **D** Immunohistochemical staining for TRPM7, SLC2A1 and SLC2A3 in tumor tissue (scale bars, 100 μm). **E**, **F** Increased SLC2A3 in naltriben-induced (25 μM, 24 h) WT cells but not TRPM7KO cells. Quantification of protein was below. Two groups of samples were compared by unpaired two-tailed Student’s *t*-test. Multiple groups were compared by ANOVA.

To pinpoint the downstream effector whereby TRPM7 controls glycolysis, we analyzed the RNA-seq data and found that TRPM7 KO significantly lowered SLC2A3 transcript (encoding the glucose transporter GLUT3), a gene among the top differentially expressed genes in TRPM7KO cells (Fig. 2A & Supplemental excel file). Furthermore, other key metabolic genes, such as HK2, ENO2 and PDK1, were also downregulated in TRPM7KO cells (Fig. 2B). This was further confirmed in vivo, as TRPM7KO tumor exhibited less SLC2A3 expression but not SLC2A1 (Fig. 2C, D). Likewise, the TRPM7 agonist naltriben elevated the mRNA and protein levels of SLC2A3 but not SLC2A1, suggesting that TRPM7 precisely regulates SLC2A3 (Fig. 2E, F). Moreover, the increased SLC2A3 expression induced by naltriben was counteracted by TRPM7 deletion, suggesting that naltriben promoting SLC2A3 was dependent on TRPM7 (Fig. 2E, F). The results collectively suggest that SLC2A3 is an unbiased target of TRPM7.

**Fig. 3:**
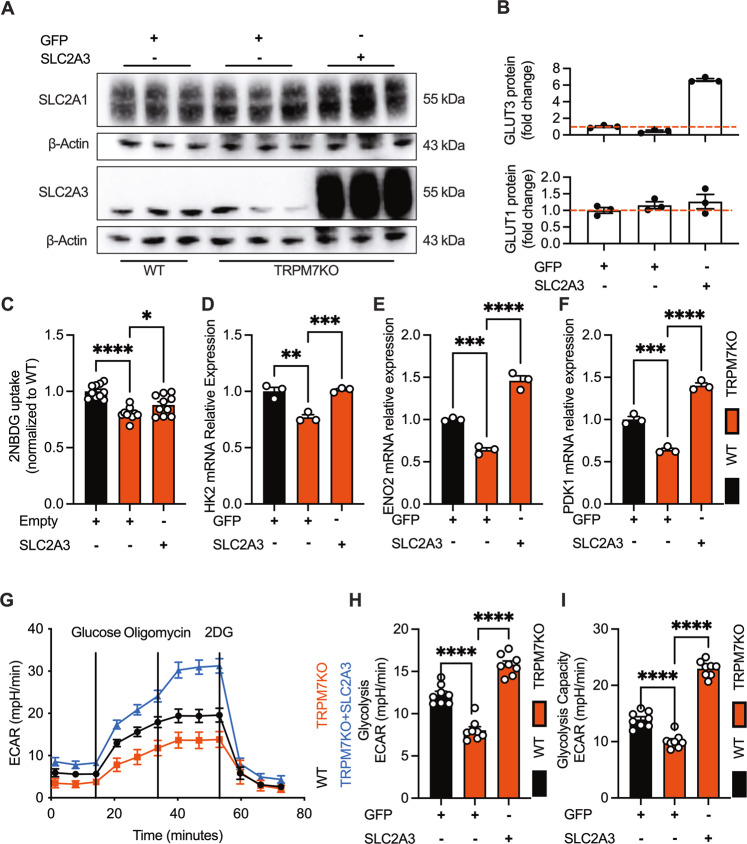
Forced expression of SLC2A3 counteracts glycolytic defect in TRPM7KO cells. **A**, **B** Western-blotting of plasma membrane TRPM7 and SLC2A3 in WT, TRPM7KO and lenti-hSLC2A3-transduced TRPM7KO cells. **C** Recovered glucose uptake in lenti-hSLC2A3-transduced TRPM7KO cells. **D**, **F** SLC2A3 transduction rescued glycolytic enzyme genes in TRPM7KO cells. **G**, **I** Recovered glycolysis in lenti-hSLC2A3-transduced TRPM7KO cells. Multiple groups were compared by ANOVA.

GLUT3, as a glucose transporter, is a known regulator of glycolysis and drives cancer cell growth [17, 18]. We further investigated whether TRPM7 controls cellular glycolysis and cell growth through SLC2A3. To gain causal link between TRPM7 and SLC2A3, we forcibly expressed SLC2A3 in TRPM7KO cells by means of hSLC2A3-encoding lentivirus, and examined glucose utilization and cell proliferation (Fig. 3A, B). As shown, forced expression of SLC2A3 in TRPM7KO cells recapitulated the phenotype of wild type cells, as evidenced by restored glucose utilization (Fig. 2C–I, S6A–D), as well as cell proliferation and migration (Fig. S7A–D). Together, the data suggest that the TRPM7- SLC2A3 axis controls cellular glycolysis and cell growth.

### TRPM7 regulates SLC2A3 transcription through Ca^2+^-induced calcineurin activation

TRPM7 is an ion channel that permeates Mg^2+^ & Ca^2+^, as well as a serine/threonine kinase. Its kinase domain has many substrates, such as annexin I, myosin IIA [19, 20]. Of interest, the kinase can be cleaved to enter into the nucleus where it can phosphorylate histones and epigenetically regulates gene transcription [11]. Thus, it is necessary to differentiate the channel or kinase functionality in glycolytic metabolism. We set up a rescue experiment where the longest cleaved kinase fragment, containing amino acids 1299–1864 (abbreviated as M7CK-L), was reconstituted in TRPM7KO cell; forced expression of MLCK-L failed to normalize SLC2A3 expression (Fig. S8A). These results imply that the cleaved kinase contributes minimally to SLC2A3 regulation. Furthermore, we attempted to restore the channel domain by forced expression of TRPM7 kinase dead mutant (K1646R) on the basis of TRPM7KO to clarify whether the channel is necessary for the regulation of SLC2A3. Unfortunately, overexpression of the channel leads to rapid cell death, which has been reported previously and may be related to ions overload (Fig. S8B) [9].

TRPM7 has been proposed as an ion channel pathway for Mg^2+^ influx, but this remains controversial [21, 22]. Given that Mg^2+^ is an indispensable co-factor of many glycolytic enzymes, it is necessary to examine whether TRPM7-mediated Mg^2+^ influx regulates glycolysis. We employed naltriben to activate the channel, and measured the intracellular free Mg^2+^ ([Mg^2+^]_i_) dynamics. In addition, we used ICP-MS to quantify the total cellular Mg^2+^. The results showed that naltriben was capable of inducing Mg^2+^ influx but this effect was still present in TRPM7KO cells (Fig. S8C). The result indicates that naltriben may exert TRPM7-independent effects to induce Mg^2+^ influx. ICP-MS assay of basal Mg^2+^ levels showed that TRPM7 KO had no effect on intracellular Mg^2+^ amounts (Fig. S8D). We also resorted to MgSO_4_ supplementation, an approach that has been shown to rescue TRPM7KO-induced cell growth defects [9]. But supplementation of TRPM7KO cells with extracellular MgSO_4_ (5 mM) was unable to normalize glucose utilization, ATP production, or SLC2A3 expression. These results suggest that Mg^2+^ is not the ion that regulates SLC2A3 transcription (Fig. S8E, F).

**Fig. 4:**
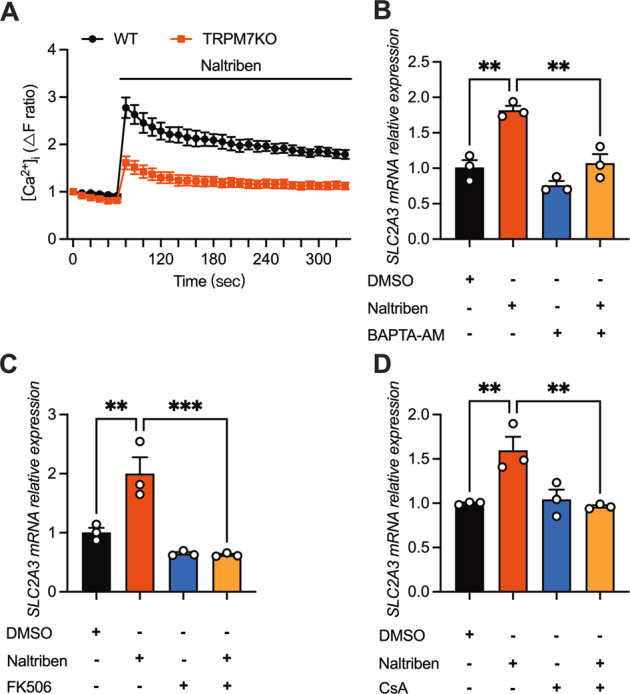
Calcineurin is the calcium effector mediating TRPM7 signaling in glycolysis. **A** [Ca^2+^]_i_ changes in WT and TRPM7KO cells induced by naltriben (25 μM) (*n* = 6). **B**–**D** Naltriben-induced (25 μM, 4 h) SLC2A3 boost counteracted by preincubation of BAPTA-AM (50 μM, 1 h), FK506 (1 μM, 1 h) or CsA (1 μM, 1 h). Multiple groups were compared by ANOVA.

TRPM7 is also a Ca^2+^-permeable channel; naltriben elicited Ca^2+^ influx, an effect that was abrogated in TRPM7KO cells (Fig. 4A). This assay indicates that the effects of naltriben are attributable to TRPM7-mediated Ca^2+^ influx. As shown, naltriben promoted SLC2A3 expression, but this effect was abolished by TRPM7 KO or chelation of intracellular Ca^2+^ with BAPTA-AM (Fig. 2F, 4B). Next, we explored the signaling pathway downstream of TRPM7-evoked Ca^2+^ entry. Block of calcineurin (a Ca^2+^-dependent phosphatase) by FK506 or CsA, rather than calpain by calpeptin or CaM kinases by KN-93, counteracted SLC2A3 expression induced by naltriben (Fig. 4C, D, S8G–H). Taken together, TRPM7 transcriptionally regulates SLC2A3 through Ca^2+^-induced calcineurin activation.

### CRTC2/CREB signaling axis acts downstream of calcineurin to control glycolytic metabolism and cell growth

**Fig. 5:**
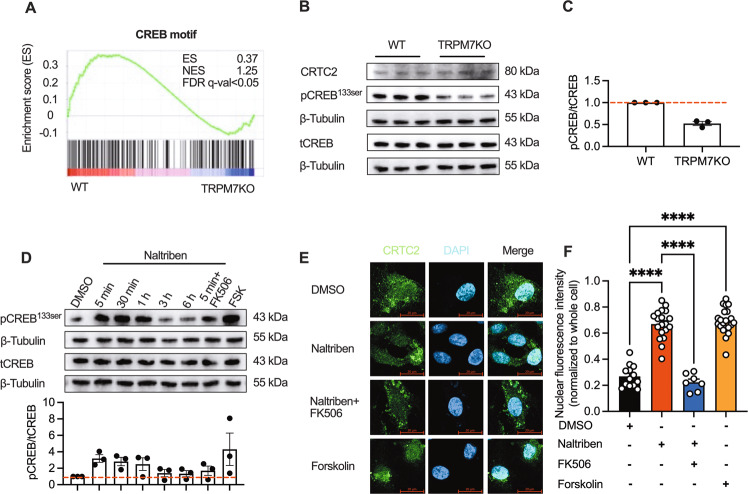
The CRTC2/CREB axis acts downstream of TRPM7/Ca^2+^/calcineurin to regulate SLC2A3 transcription. **A** GSEA of the CREB DNA-binding element gene sets in WT or TRPM7KO cells. **B**, **C** Immunoblot analysis of CREB signaling in WT and TRPM7KO cells. **D** Immunoblot analysis of CREB signaling in response to naltriben (25 μM), FK506 (preincubation, 1 μM) or forskolin (10 μM). **E**, **F** Nuclear translocation of CRTC2 in response to naltriben (25 μM, 2 h), FK506 (preincubation, 1 μM, 1 h) or forskolin (10 μM, 2 h) (scale bars, 20 μm). Two groups of samples were compared by unpaired two-tailed Student’s *t*-test. Multiple groups were compared by ANOVA.

**Fig. 6:**
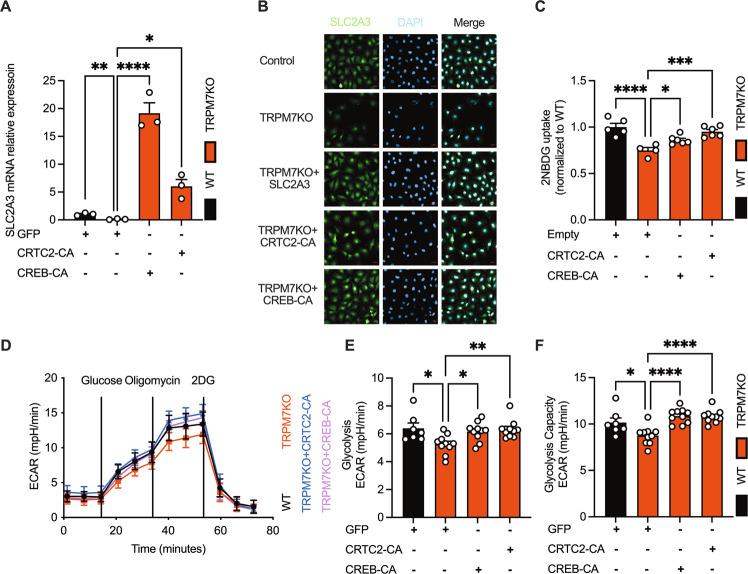
Restoration of CRTC2 and CREB rescues impaired SLC2A3-mediated glycolytic defects in TRPM7KO cells. **A**, **B** Enhanced SLC2A3 transcripts under the forced expression of SLC2A3, CRTC2-CA (Tyr134Phe) or CREB-CA (Ser171Ala, Ser275Ala) (scale bars, 20 μm). **C**–**F** Normalized glucose uptake and glycolysis in lenti-hCREB-CA or lenti-hCRTC2-CA-transduced TRPM7KO cells. Multiple groups were compared by ANOVA.

The KEGG analysis pointed to enrichment of cAMP signaling pathway in TRPM7KO cells (Fig. S3B). GSEA showed that the CREB DNA-binding motif was highly enriched in the repressed genes upon TRPM7 KO (Fig. 5A). CREB phosphorylation was markedly repressed by TRPM7 KO (Fig. 5B). The promoter of SLC2A3 gene can bind to the transcription factor CREB, which is regulated by its transcriptional coactivator CRTC2 [23]. All the evidence indicates that CRTC2/CREB axis may link TRPM7 channel to glycolytic metabolism. Dephosphorylation of CRTC2 renders it to enter into the nucleus where it binds to and phosphorylate CREB. CRTC2 are regulated by 2 pathways: cAMP/PKA & calcineurin [24]. Of note, naltriben increased intracellular cAMP level and promoted PKA phosphorylation (Fig. S9A, B). The effects of naltriben could be abolished by the calcium chelator-BAPTA, indicating that TRPM7-mediated Ca^2+^ entry contributed to PKA activation (Fig. S9B). Also, forskolin, an agonist of cAMP/PKA, could rescue the proliferative defect mediated by TRPM7KO (Fig. S9C). Forskolin only partially rescued reduced SLC2A3 in TRPM7KO cells (Fig. S9D), and TRPM7KO did not affect PKA phosphorylation (Fig. S9E). Nonetheless, calcineurin inhibitors completely blocked naltriben-driven SLC2A3 rise (Fig. 4C, D). Consequently, we speculated that cAMP/PKA axis may not be the major pathway for TRPM7 to regulate SLC2A3. Then, we checked whether CRTC2 is the primary target of TRPM7-calcineurin. As shown, naltriben elevated CREB phosphorylation as well as CRTC2 nuclear translocation, an effect that was blocked by the calcineurin inhibitor - FK506 (Fig. 5D–F). These results suggest that TRPM7 channel activates CRTC2/CREB via calcineurin. Finally, the constitutively active mutants of CRTC2 (CRTC2-CA) or (CREB-CA) were expressed in TRPM7KO cells to preserve CRTC2/CREB activity in the absence of TRPM7 activity. Of note, CRTC2-CA or CREB-CA normalized TRPM7 deficiency-induced defects in tumor growth, glucose consumption, as well as SLC2A3 expression (Fig. 6A–F, S9A–D, S10A–D). Hence, these data suggest that CRTC2/CREB relays TRPM7-mediated Ca^2+^ signal to SLC2A3 transcription.

### TRPM7 is a conserved regulator of EC growth, glycolytic metabolism and retinal angiogenesis

**Fig. 7:**
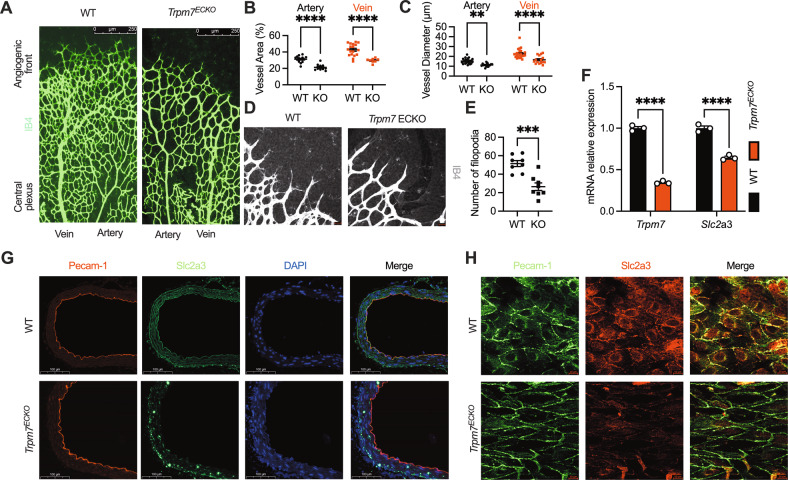
Endothelial TRPM7 deletion in vivo impairs postnatal retinal vessel growth. **A** Images of IB4-stained P 6.5 retinas of WT and *Trpm7*^*ECKO*^ mice (scale bars, 250 μm, *n* ≥ 6 mice/group). **B**, **C** Quantification of vascular parameters in WT and *Trpm7*^*ECKO*^ mice. **D**, **E** Quantification of filopodia in WT and *Trpm7*^*ECKO*^ mice. **F** RT-qPCR of endothelial *Slc2a3* in mouse lung endothelial cells. **G** Decreased expression of aortic endothelial GLUT3 (*Slc2a3*) in *Trpm7*^*ECKO*^ mice (scale bars, 100 μm, *n* ≥ 4 mice/group). **H** Confocal immunofluorescent microscopy of endothelial Slc2a3 in the en-face aortic endothelium in WT and *Trpm7*^*ECKO*^ mice (scale bars, 10 μm, *n* ≥ 4 mice/group). Two groups of samples were compared by unpaired two-tailed Student’s *t*-test.

Having demonstrated TRPM7 as a regulator of cancer cell glycolysis, we further characterized this channel in healthy VECs that are analogous to cancer cells in cellular metabolism. In the cardiovascular system, TRPM7 has been implicated in atrial fibrillation, cardiac remodeling, vascular smooth muscle phenotypic switching, vascular calcification [13–15]. However, the functional role of endothelial TRPM7 remains incompletely appreciated and controversial. To obtain rigorous proof, we generated EC-specific TRPM7-KO mice (*Trpm7*^*ECKO*^) by breeding *Trpm7*^*fl/fl*^ mice with *Tie2-Cre* mice. The *Trpm7*^*ECKO*^ mice are embryonically viable and grow postnatally without gross abnormality (Fig. S11). This mouse line enabled exploration of postnatal blood vessel growth. Of note, isolectin-B4 (IB4) immunofluorescence examination of postnatal retinal vessel growth revealed a sparse vascular network in *Trpm7*^*ECKO*^ mice (Fig. 7A). Additionally, the narrower vessel diameter and fewer filopodia were observed in *Trpm7*^*ECKO*^ mice, indicating that endothelial *Trpm7* is critical for postnatal vascular development (Fig. 7B–E). We also isolated mouse lung ECs (mLECs) and quantified the SLC2A3 transcript. As revealed, SLC2A3 was downregulated in mLECs derived from *Trpm7*^*ECKO*^ mice (Fig. 7F). Additionally, endothelial plasmalemmal SLC2A3 expression was reduced in *Trpm7*^*ECKO*^ mice, as shown by immunostaining of aortic endothelium (Fig. 7G–H). The result suggests that TRPM7 is required for SLC2A3 in endothelial cells in vivo.

**Fig. 8:**
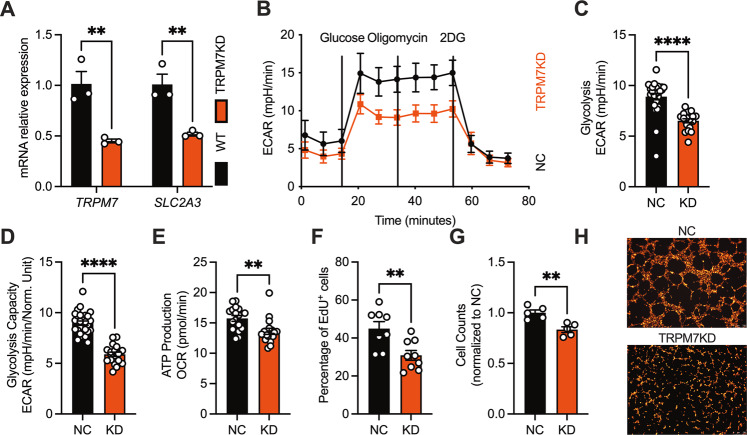
The TRPM7 channel tunes endothelial glycolysis and cell growth in vitro via SLC2A3. **A** RT-qPCR of SLC2A3 in TRPM7 siRNA- or NC-transfected human umbilical vein endothelial cells (HUVECs). **B**–**E** Defective glycolysis and ATP production in TRPM7 knockdown (KD) HUVECs. **F**–**G** Decreased cell counts and suppressed cell cycle in TRPM7KD HUVECs. **H** Impaired tube formation in TRPM7KD HUVECs staining with lipophilic carbocyanine dye (scale bars, 250 μm). Two groups of samples were compared by unpaired two-tailed Student’s *t*-test.

**Fig. 9:**
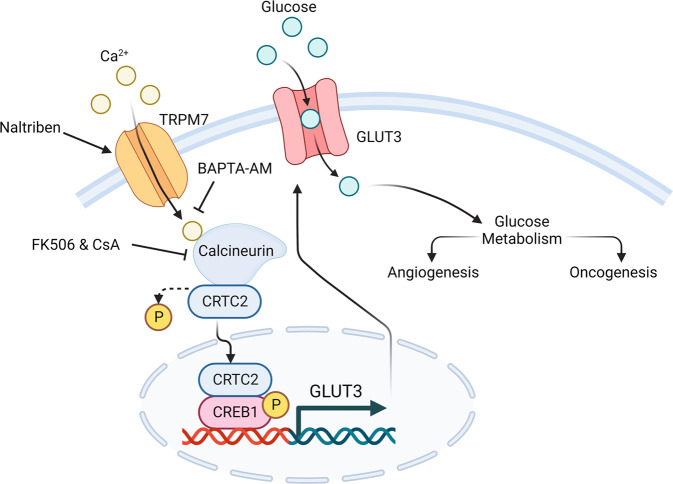
TRPM7-SLC2A3 axis is conserved across cancer and endothelial cells. TRPM7 regulates SLC2A3 to drive tumorigenesis and angiogenesis via ca^2+^/calcineurin/CRTC2/CREB axis.

In human umbilical vein endothelial cells (HUVECs), knockdown of TRPM7 suppressed cell growth, tube formation, accompanied by reduced glucose utilization and SLC2A3 expression (Fig. 8A–H, S12A–D). Forced expression of SLC2A3 in TRPM7-knockdown HUVECs promoted endothelial growth, glucose uptake and ATP production (Fig. S12E–H). These data converge to suggest that the TRPM7-SLC2A3 axis is conserved across cancer and endothelial cells (Fig. 9).

## Discussion

The findings presented here unveil a conserved, previously unrecognized mechanism whereby the TRPM7 channel drives oncogenesis and angiogenesis. We have demonstrated that TRPM7 is a novel regulator of glycolytic reprogramming. While our study is underway, a most recent study employed TRPM7 knockdown approach for in vitro assays, and reported that TRPM7 knockdown dampened ovarian cancer cell glycolysis via AMPK activation and HIF1 degradation [25]. Our study, however, relies on TRPM7 knockout for an integrated set of in vitro & in vivo experiments, and found that the TRPM7/calcineurin/CRTC2/CREB/SLC2A3 axis controls cellular glycolysis. One notable strength of our study is the differentiation of TRPM7 channel or kinase during mechanistic interrogation and the dissection of the signaling transduction pathway. Of interest, we found that TRPM7 KO suppresses both glycolysis and oxidative phosphorylation in T24 & HUVECs, in contrast to the observation that TRPM7 knockdown inhibited glycolysis but enhanced TCA in ovarian cancer cell. This discrepancy may result from the fact that ovarian cancer cell prefers glutaminolysis over glycolysis for tumor growth [16, 26]. In this study, we attempted to extrapolate our cancer cell findings to endothelial cell. However, the role of TRPM7 in endothelial growth is still controversial. TRPM7 silencing by siRNA promoted endothelial migration and proliferation, but also paradoxically impaired endothelial proliferation and viability [27, 28]. The experimental conditions may underlie the controversy. To offer solid evidence, we used *Trpm7*^*ECKO*^ mice to investigate the in vivo role of endothelial TRPM7. Of significance, deletion of endothelial TRPM7 impaired retinal vessel growth, and also reduced SLC2A3 expression in mouse pulmonary or aortic endothelial cells. In agreement, TRPM7 knockdown in HUVECs impaired endothelial growth and tube-formation in vitro. Hence, we have provided reliable evidence that TRPM7 regulates endothelial physiology.

Intracellular Ca^2+^ signaling is known to drive metabolic reprogramming, but the exact ion channel that perturbs [Ca^2+^]_i_ has yet to be unveiled. This study suggests that TRPM7, unlike other Ca^2+^-permeable channels, is an ion channel that serves as a metabolic rheostat. TRPM7 is a channel-kinase chimera, but our data exclude the kinase as the functional domain regulating cellular glycolysis. Using channel opener and Ca^2+^ signaling blockers, we showed that TRPM7 transcriptionally regulates SLC2A3 via Ca^2+^ rather than Mg^2+^ signals. One caveat of this study is the use of small molecule TRPM7 opener rather than endogenous TRPM7 activator. TRPM7 is potently blocked by cytosolic Mg^2+^ or Mg∙ATP, and is also modulated by mechanical stretch [9, 29, 30]. The mechanosensitivity of TRPM7 channel has been demonstrated in VSMCs and cancer cells [29, 30]. Indeed, mechanical stress triggers SLC2A3-mediated glycolytic burst to drive VECs motility [31]. Fluid shear stress is required for endothelial function and vascular development [32, 33]. Using human or mouse-derived healthy and tumor cells, we uncovered that TRPM7 regulates SLC2A3 in a conserved manner. Notably, retinal vessel growth is defective upon endothelial TRPM7 deletion. Future studies may determine whether mechanical stress impacts cellular glycolysis and function through the TRPM7/SLC2A3 axis.

CRTC2 and CREB have been well documented in hepatic gluconeogenesis, but their role in glucose catabolism is understudied [34, 35]. Our data have validated that TRPM7 deficiency reduces tumor and EC glycolytic metabolism by inactivating the CRTC2/CREB axis. This would open the door for further understanding the CRTC2/CREB axis in glucose homeostasis. We found that Ca^2+^/calcineurin, rather than Mg^2+^/kinase, links TRPM7 channel function to CRTC2. It is noteworthy that TRPM7 deletion inactivates calcineurin without reducing the basal [Ca^2+^]_i_. This may be owing to the fact that TRPM7 channel opening results in a localized, rather than global increase in Ca^2+^. It has been observed that Ca^2+^ proximal to TRPM7 channel pore, instead of global cytosolic Ca^2+^ mediates lipopolysaccharide-induced TLR4 endocytosis [36]. We propose that TRPM7 channel opening generates a Ca^2+^ “hot spot” for calcineurin activation, but this warrants further validation.

In summary, the present study links the TRPM7 chanzyme to cellular glycolytic metabolism and untangles the TRPM7/calcineurin/CRTC2/CREB/SLC2A3 axis as the mechanism. The data offer novel insights into regulation of cellular glycolysis by ion channel, and suggest that the TRPM7 channel is an exploitable target for cancer therapy and pathological neovascularization.

## Supplementary information

checklist

sup info

Dataset 1

## Data Availability

The RNA-seq data that supports the findings of this study are available in the supplementary material of this article.
