# Supplementary material for: The TRPM7 channel reprograms cellular glycolysis to drive tumorigenesis and angiogenesis
Source: Cell Death Dis. 2023 Mar 6;14(3):183. doi: 10.1038/s41419-023-05701-7 (PMC9988972; doi:10.1038/s41419-023-05701-7)
Supplement: Supplementary file 2 — sup info [file 41419_2023_5701_MOESM2_ESM.docx]

**SUPPLEMENTAL INFORMATION**

**The TRPM7 Channel Reprograms Cellular Glycolysis to Drive Tumorigenesis and Angiogenesis**

Wanzhou Wu ^1, 2, 3*^, Xuan Wang ^3*^, Longsheng Liao ^3^, Jing Chen ^3^, Yue Wang ^3^, Meilian Yao ^3^, Lingping Zhu ^3^, Jiayu Li ^4^, Xuan Wang ^5^, Alex F Chen ^2, 6^, Guogang Zhang ^3, 7^, Zheng Zhang ^5^, Yongping Bai ^3, 7^

^1^ Department of Cardiology, The Third Xiangya Hospital, Central South University, Changsha, China.

^2^ Center for Vascular Disease and Translational Medicine, Department of Cardiology, The Third Xiangya Hospital, Central South University, Changsha, China.

^3^ Department of Geriatric Medicine, Xiangya Hospital, Central South University, Changsha, China.

^4^ College of Pharmacy, Hunan University of Chinese Medicine, Changsha, China.

^5^ Department of Pharmacology, Hunan Key Laboratory of Cardiovascular Research, Xiangya School of Pharmaceutical Sciences, Central South University, Changsha, China.

^6^ Institute of Development and Regenerative Medicine, Xinhua Hospital Affiliated to Shanghai Jiaotong University School of Medicine, Shanghai, China.

^7^ National Clinical Research Center for Geriatric Disorders, Xiangya Hospital, Central South University, Changsha, China.

^*^ These authors contributed equally to this work.

**Corresponding author:**

Yongping Bai, MD, PhD, Department of Geriatric Medicine, Xiangya Hospital, Central South University, No. 87 Xiangya Road, Changsha 410008, China. E-mail: [baiyongping@csu.edu.cn](mailto:baiyongping@csu.edu.cn)

Zheng Zhang, MD, PhD, Department of Pharmacology, Xiangya School of Pharmaceutical Sciences, Central South University, No. 110 Xiangya Road, Changsha 410078, China. E-mail: zzhang@csu.edu.cn

**SUPPLEMENTAL METHODS**

**2.1 *Trpm7* knockout (KO) mice.** *Trpm7^fl/fl^* mice were purchased from the Jackson Laboratory. To generate endothelium-selective deficiency of *Trpm7 (Trpm7^ECKO^)*, *Trpm7^fl/fl^* mice were crossed with *Tie2-Cre* mice. *Cre*-positive male mice were used for intercrossing to prevent recombination in the female germline. Animal experiments were performed in accordance with guidelines and protocols approved by the Ethics Committee of Xiangya Hospital.

**2.2 Retinal vessel growth analysis.** As previously reported [1], whole eyes from mice on postnatal day (P) 6.5 were fixed in 4% paraformaldehyde for 1 h at room temperature (RT). After blocking and permeabilization in retina-blocking buffer (1% bovine serum albumin [BSA] and 0.5% Triton X-100 in PBS) overnight, the retinas were incubated at 4 ℃ in PBLEC buffer (1 mM CaCl_2_, 1 mM MgCl_2_, 0.1 mM MnCl_2_, and 1% Triton X-100 in PBS) with Alexa Fluor 488-conjugated IB4 (Thermo Fisher, I21411, 1:200) overnight. After washing with PBLEC buffer, the retinas were dissected into four leaflets. DAPI Fluoromount-G (Southern Biotech, 0100-20) was used for nuclear counterstaining. The retinas were examined by confocal laser microscopy (Zeiss LSM 900 with Airyscan2).

**2.3 Subcutaneous tumor xenograft model.** BALB/c-nu mice were purchased from Hunan SJA Laboratory Animal Co., Ltd. (Changsha, China). Cells (5×10^6^) were diluted in 200 µL of PBS and Matrigel. Tumors were evaluated and recorded regularly after cell transplantation. The volume of the tumors was measured as follows: V = D×d^2^×π/6, where V is volume, D is the longest diameter of the bulk tumor, and d is the shortest diameter of the bulk tumor. No mice died during the experiment.

**2.4 Cell culture.** Primary HUVECs were gained from ScienCell and cultured in ECM (ScienCell, 1001) supplemented with EC growth supplement (ECGS), 5% fetal bovine serum (FBS), and a penicillin/streptomycin cocktail. HUVECs were used for analysis before the 5th passage. mLECs were isolated as described. In brief, the lungs of adult mice were harvested and incubated with dispase (Roche, 10269638001). The homogenate was filtered through 100-μm and 40-μm cell strainers. The cell suspension was collected and incubated with mouse CD31 microbeads (Miltenyi Biotec, 130-097-418). The beads were washed with Dulbecco’s PBS (DPBS) supplemented with 1% FBS and then used for total RNA isolation. HEK293T cells were gained from the National Collection of Authenticated Cell Cultures and cultured in DMEM supplemented with 10% FBS. Wild type and TRPM7 KO T24 human urinary bladder carcinoma cells were generated in Dr. Zheng Zhang’s lab. The cells were authenticated through short tandem repeat (STR) by Genetic Testing Biotechnology Corporation (Suzhou, China) and cultured in DMEM supplemented with 10% FBS.

**2.5 Immunohistochemistry.** Cells were seeded on glass coverslips at 37 ℃ with 5% CO_2_. After removing the cell culture medium and washing with PBS, the cells were fixed with 4% paraformaldehyde for 15 min at room temperature. The cells were washed with 0.03% Triton X-100 in PBS, and permeabilizations were performed in 3% donkey serum and 0.1% Triton X-100 in PBS. The cells were stained with primary antibodies at 4 ℃ overnight. After removing primary antibodies and washing with PBST (0.1% Triton X-100 in PBS), the samples were incubated with Alexa Fluor-conjugated secondary antibodies (Invitrogen, 1:200) at RT for 1 h. DAPI Fluoromount-G was used for nuclear counterstaining. All samples were examined by confocal laser microscopy (Zeiss LSM 900 with Airyscan2).

**2.6 Bulk RNA sequencing.** The RNA-sequencing and bioinformatics analysis was performed by Metware (Wuhan). In brief, total RNA was lysed with RNAiso Plus (TaKaRa, 9109). The contamination and degradation of RNA were inspected on 1% agarose gels. The NanoPhotometer spectrophotometer (IMPLEN) and Qubit 2.0 fluorometer (Life Technologies) with Qubit RNA assay kit were used to determine RNA purity and concentration respectively. Bioanalyzer 2100 system (Agilent Technologies) with RNA Nano 6000 assay kit was used for assessing RNA integrity. The RNA Integrity Numbers were over 6.8 in the study samples. The NEBNext Ultra RNA Library Prep Kit was used for generating libraries. After cluster generation, the constructed library was subjected to 125-bp/150-bp paired-end reads on Illumina HiSeq platform. Fastq files were processed with in-house Perl scripts. Low-quality reads were removed to generate clean data and subsequent analysis. HISAT2 (v2.0.5) was used for building reference genome index and aligning paired-clean reads. FeatureCounts (v1.5.0-p3) was used for counting read numbers. Calculating FPKM based on the length and read counts of each gene. Differential expression of two groups was analyzed by DESeq2 R package (v1.16.1) and *P*<0.05 was considered to be statistically differential. Benjamini and Hochberg method were used to correct *P* value. Differential genes should meet both corrected *P*<0.05 and fold change>2. The data that supports the findings of this study are available in the supplementary material of this article.

**2.7 Transfection & Lentiviral transduction.** The siRNAs were transfected into HUVECs through Nucleofector 2b (Lonza) with an Amaxa basic nucleofector kit for primary mammalian ECs (Lonza, VPI-1001) according to the manufacturer’s instructions. The lentivirus encoding constitutively active CRTC2, constitutively active CREB, SLC2A3 and an empty vector for *in vitro* experiments was generated and amplified in HEK293T cells. The virus was concentrated by a Lenti-X Concentrator (Clontech, 631231). The following plasmids were used in this study: pLenti-mCrebCA, pLenti-mCrtc2CA, pLenti-hSLC2A3, and pCMV-M7CK-L.

**2.8 Reagents.** Basic fibroblast growth factor (FGF2, 233-FB-010) was purchased from R&D Systems. Vascular endothelial growth factor (VEGF, 100-20) was purchased from PeproTech. Ionomycin calcium salt (1704) and cyclosporin A (1101) were purchased from TOCRIS. Magnesium sulfate (M2643), heparin (H3194) and paraformaldehyde (158127) were obtained from Sigma–Aldrich. BAPTA-AM (HY-100545), forskolin (HY-15371) and FK506 (13756) were purchased from MedChemExpress. Matrigel Matrix (354230) was obtained from Corning.

**2.9 Quantitative reverse transcription PCR.** Total RNA was isolated with RNAiso Plus (TaKaRa, 9109). Reverse transcription and subsequent quantitative PCR were performed with PrimeScript RT reagent kit with gDNA Eraser (TaKaRa, RR047) and TB Green Premix Ex Taq (TaKaRa, RR420) on a QuantStudio Dx Real-Time PCR instrument (Thermo Fisher). The reaction was started with a denaturation step at 95 ℃ for 30 s followed by 40 cycles of denaturation at 95 ℃ for 5 s and annealing and elongation at 60 ℃ for 34 s. Fluorescence data were collected at the end of each cycle. The comparative Ct method (△△Ct) was used for analyzing the data. All transcript of genes was corrected by ACTB.

**2.10 Western blotting.** Total protein lysates were collected in ice-cold RIPA buffer containing protease and phosphatase inhibitors. A bicinchoninic assay (BCA) kit (Beyotime Biotechnology, P0011) was used for protein quantification. The protein lysates were separated by SDS–PAGE and transferred to PVDF membranes (Millipore, IPVH00010). After blocking in 5% nonfat powdered milk (Sangon Biotech, A600669) or 5% BSA, the membranes were washed with TBST three times and incubated overnight at 4 ℃ with primary antibody. The membranes were incubated with horseradish peroxidase (HRP)-conjugated secondary antibodies (Abcam, ab6721) for 1 h at room temperature followed by chemiluminescence detection with Immobilon Crescendo Western HRP substrate (Millipore, WBLUR0500). The images were generated using ChemiDoc XRS+ (Bio–Rad) with Image Lab software. The following primary antibodies were used: anti-TRPM7 (Thermo Fisher, PA5-102835, 1:500), anti-GLUT3 (Thermo Fisher, MA5-32697, 1:1000), anti-GLUT1 (Thermo Fisher, PA5-16793, 1:1000), anti-CREB (Cell Signaling Technology, 9197S, 1:1000), anti-phosphorylated CREB^Ser133^ (Cell Signaling Technology, 9198S, 1:1000), anti-CRTC2 (Thermo Fisher, MA5-15710, 1:1000), anti-ACTB (Cell Signaling Technology, 4790S, 1:1000), anti-β Tubulin (Abcam, ab6046), anti-PKA α/β/γ (AiFang, AF03120), anti-phosphorylated PKA Thr197 (AiFang, AF14521).

**2.11 Cell count analysis.** Cell counts was determined by Cell Counting Kit-8 (CCK-8) assay, Dojindo, CK04). In brief, ECs were resuspended in complete medium and seeded on a 96-well plate. After incubation for 24 h at 37 °C with 5% CO_2_, CCK-8 buffer was added and further incubated for 2 h. The absorbance was measured at 450 nm with a Cell Imaging Multi-Mode reader (BioTek, Cytation 5). All the data were compared with the control group after the background absorbance was subtracted.

**2.12 Cell proliferation.** EdU staining (Ribo Bio, C10310-3) was used for assaying cell proliferation. In general, ECs were seeded on a 96-well plate and futher incubated for 24 h at 37 °C with 5% CO_2_. EdU staining was diluted with medium at 1:1000 and added to cells growing 96-well plates. After incubation at 37 °C with 5% CO_2_ for 2 h, removing EdU and washing cells with PBS at least two times. Next, the cells were fixed with 4% paraformaldehyde for 30 min. Then, the paraformaldehyde was removed, and 2 mg/mL glycine was added and incubated for 5 min. After being washed with PBS three times, 0.5% Triton X-100 was added and incubated for 10 min. The cells were incubated with Apollo stain for 30 min and washed with 0.5% Triton X-100 three times. The data were collected with a fluorescence microscope (Leica, DMi8).

**2.13 Wound healing migration assays.** HUVEC migration was assessed in a wound healing migration using culture inserts (ibidi, 80209).

**2.14 Tube formation assay.** Tube formation of HUVECs was performed by a μ-slide angiogenesis (ibidi, 81506). DiI stain (Sigma–Aldrich, 42364) was used for labelling the cell membrane.

**2.15 Lactate assay.** Intracellular and extracellular lactate levels were assessed with a Lactate Assay kit (Abcam, ab65331). In brief, ECs were washed with cold PBS and resuspended in lactate assay buffer. The samples were centrifuged for 5 min at 4 ℃ at top speed in a cold microcentrifuge, and the supernatant was then collected in a clean tube. Then, the reaction mixture was prepared with agents according to the manufacturer’s instructions and added to the sample wells in a 96-well plate and incubated for 30 min. The lactate level was measured with a Cell Imaging Multi-Mode reader (BioTek, Cytation 5) at 450 nm.

**2.16 Glucose uptake analysis.** Glucose uptake was measured using 2-NBDG (Invitrogen, N13195). The calculation of glucose consumption efficiency was quantified by testing the residual glucose in the medium at a specific time and comparing it with the initial glucose level.

**2.17 Cell invasion.** The invasion ability was measured by transwell (Corning) analysis. In brief, cells were resuspended in serum-free medium and added to the upper compartment, in the presence of 20% FBS medium in the lower compartment. After incubation for 24 hours, cells in the lower compartment were fixed by 4% PFA and stained with crystal violet and further counted for analysis.

**2.18 ^13^C-Metabolic flux.** The ^13^C-metabolic flux was supported by BioNovoGene (Suzhou). T24 cells were cultured in DMEM with 50% D-glucose-^13^C_6_ (Sigma–Aldrich, 389374) and 50% D-glucose-^12^C_6_ for 6 h. Further, cells were separated with 0.25% trypsin-EDTA (Sigma–Aldrich, T4049) and resuspended in 0.6 mL of cold (-40 °C) 50% aqueous methanol containing 100 μM norvaline as an internal standard, placed on dry ice for 30 min, and thawed on ice. Then, 0.4 mL of chloroform was added and vortexed for 30 seconds before centrifugation (4 °C, 14000 rpm, 10 min). The supernatant was transferred to new tubes, evaporated and stored at -80 °C before analysis. Then, metabolites were derivatized for gas chromatography (GC)/mass spectrometry (MS) analysis. First, 70 μL of pyridine was added to the dried cell pellet and incubated (80 °C, 20 min). After cooling, 30 μL of N-tert-butyldimethylsilyl-N-methyltrifluoroacetamide (Sigma–Aldrich) was added, and the samples were incubated (80 °C, 60 min) before centrifugation (4 °C, 14000 rpm, 10 min). The supernatant was transferred to an autosampler vial for GC/MS analysis. A Shimadzu QP-2010 Ultra GC–MS system was programmed with an injection temperature of 250 °C, and then, 1 μL of sample was injected. The GC oven temperature was 110 °C for 4 min, increasing to 230 °C at a 3 °C/min rate and to 280 °C at a 20 °C/min rate and then held at this temperature for 2 min. The GC flow rate with helium carrier gas was 50 cm/s. The GC column used was a 20 m × 0.25 mm × 0.25 mm Rxi-5ms. The GC–MS interface temperature was 300 °C, and the electron impact ion source temperature was set at 200 °C with a 70 V ionization voltage. The mass spectrometer was set to a scan range of m/z 50-800 with a 1 kV detector. The GC/MS data were analyzed to determine isotope labeling and quantities of the metabolites. Metabolites with baseline separated peaks were quantified on the basis of the total ion count peak area using standard curves generated from standards included in the same batch as the samples. To evaluate the ^13^C labeling, the mass distribution for fragments of known metabolites was extracted from the appropriate chromatographic peak. These fragments contained either the whole carbon skeleton of the metabolite, lacked the alpha carboxyl carbon, or (for some amino acids) contained only the backbone without side chain 1. For each fragment, the retrieved data comprised mass intensities for the lightest isotopomer (without y heavy isotopes, M0) and isotopomers with increasing unit mass (up to M6) relative to M0. These mass distributions were normalized and corrected for the natural abundance of heavy isotopes of the elements H, N, O, Si and C using matrix-based probabilistic methods as previously described2, and the data were evaluated in MATLAB. The labeling results are expressed as a fraction of the particular compound that contains isotopic labels of a specific precursor.

**2.19 Seahorse analysis.** Cell glycolysis and respiration metabolism were measured on an XF^e^96 extracellular flux analyzer (Agilent Technologies, CA, USA) with a Seahorse XF glycolysis stress test kit (Agilent, 103020-100) and Cell Mito stress test kit (Agilent, 103015-100). For the glycolysis test, the assay medium was XF DMEM (Agilent, 103015-100) supplemented with 2 mM L-glutamine (Life Technologies, 25030-081), and the following agents were added at the stated concentrations: 10 mM glucose, 1 μM oligomycin and 50 mM 2-DG. For the Mito stress test, the assay medium was XF DMEM supplemented with 2 mM L-glutamine, 1 mM pyruvate and 10 mM glucose. The following agents were used at the stated concentrations: 1.5 μM oligomycin, 1 μM fluoro-carbonyl cyanide phenylhydrazone (FCCP) and 1 μM rotenone/and antimycin A (Rot/AA). The ECAR and OCR were normalized on the basis of the cell counts according to the manufacturer’s recommendation. The glycolysis rate, glycolytic capacity, reverse glycolysis rate, basal respiration rate, maximal respiration rate and ATP production level were calculated according to the Seahorse kit instructions.

**2.20 Intracellular calcium and magnesium measurements.** Cells were seeded into a 96-well plate and incubated with Fluo-4AM (Invitrogen, F14201) or Magnesium-Green AM (Invitrogen, M3735) supplemented with 0.05% pluronic F-127 (Sigma–Aldrich, P2443) for 30 min at 37 ℃ followed by a 30 min wash with Hank’s balance salt solution (Sigma–Aldrich, H8264) at room temperature. The fluorescence was measured using a Cell Imaging Multi-Mode reader (BioTek, Cytation 5).

**2.21 Inductively coupled plasma (ICP)–MS.** The basal level of intracellular magnesium was measured by ICP–MS (Agilent, 7700). The experimental protocols were followed as described in previous studies [2].

**2.22** **cAMP detection. The intracellular cAMP level was measured by cAMP-Glo Assay kit (Promega, V1501).** Cells (1×10^4^) were seeded into a 96-well plate and cultured overnight. Then, cells were incubated with PBS or naltriben (25 μM in PBS) for 5 min. Subsequently, add the lysis buffer, cAMP detection solution and Kinase-Glo Reagent to all wells according to the protocol of Promega. The relative luminescence unit was detected by a Cell Imaging Multi-Mode reader (BioTek, Cytation 5). The standard curve was used to analyze intracellular cAMP levels.

**2.23 Statistical analysis.** Statistical analysis was performed with GraphPad Prism software (v. 9.2.0). All experimental values are presented as the mean ± s.e.m. Statistical significance was assessed by ANOVA or Student’s *t* test. A *P* value <0.05 was considered to be statistically significant. All studies were repeated by two independent experimenters at least three times. No randomization or blinding was enforced, and no animals were eliminated from the analyses.

**References**

1. Wilhelm K, Happel K, Eelen G, Schoors S, Oellerich MF, Lim R, et al. FOXO1 couples metabolic activity and growth state in the vascular endothelium. Nature. 2016;529(7585):216-20. doi: 10.1038/nature16498.

2. Jin J, Desai BN, Navarro B, Donovan A, Andrews NC, Clapham DE. Deletion of Trpm7 disrupts embryonic development and thymopoiesis without altering Mg2+ homeostasis. Science. 2008;322(5902):756-60. doi: 10.1126/science.1163493.

**Supplementary Figures and Figure Legends**

**
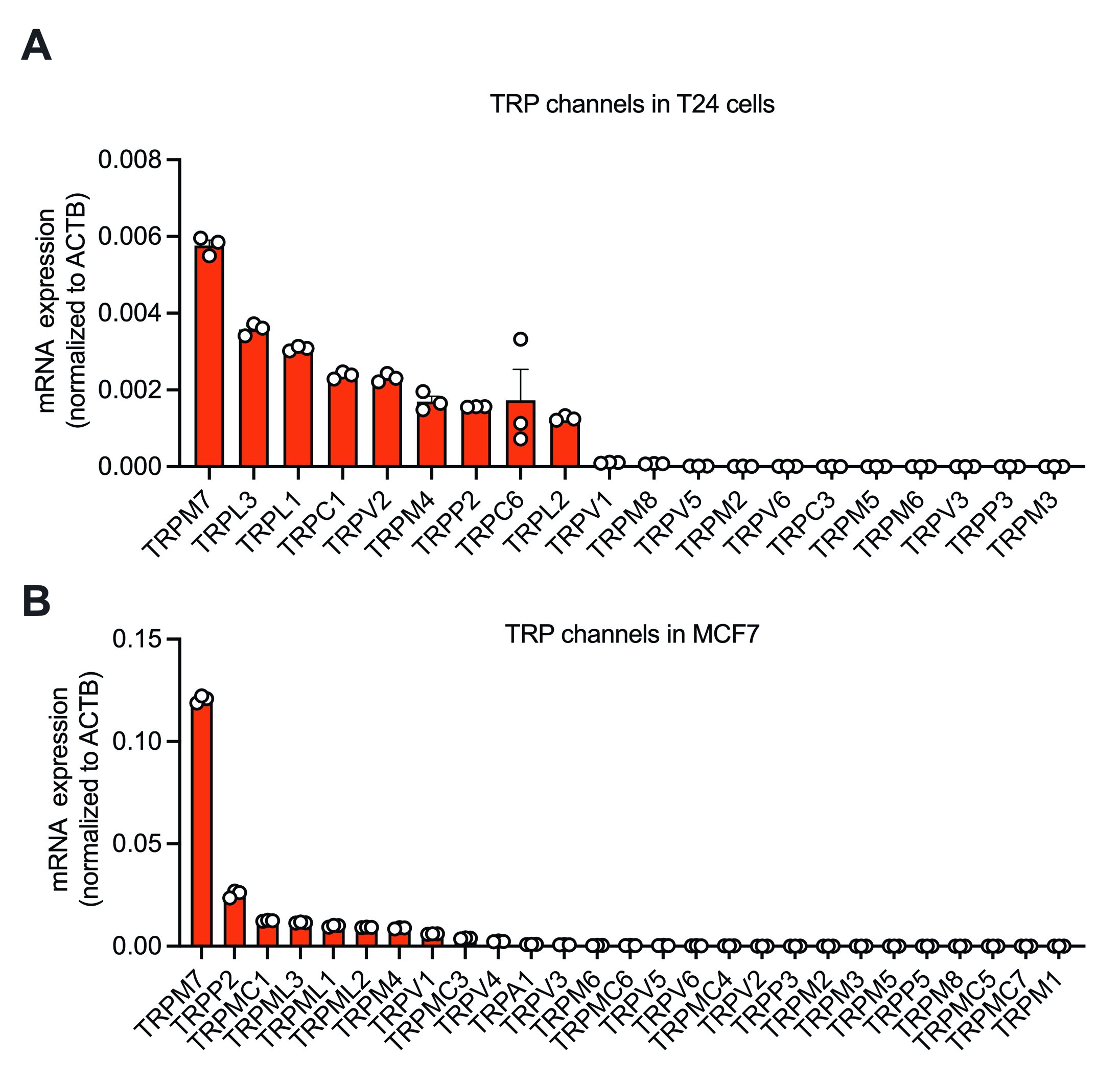
**

**Figure S1.** **Expression of TRPM7 in diverse cell types.**

**A-B.** Expression of TRP channel family in T24 and MCF7 cells.


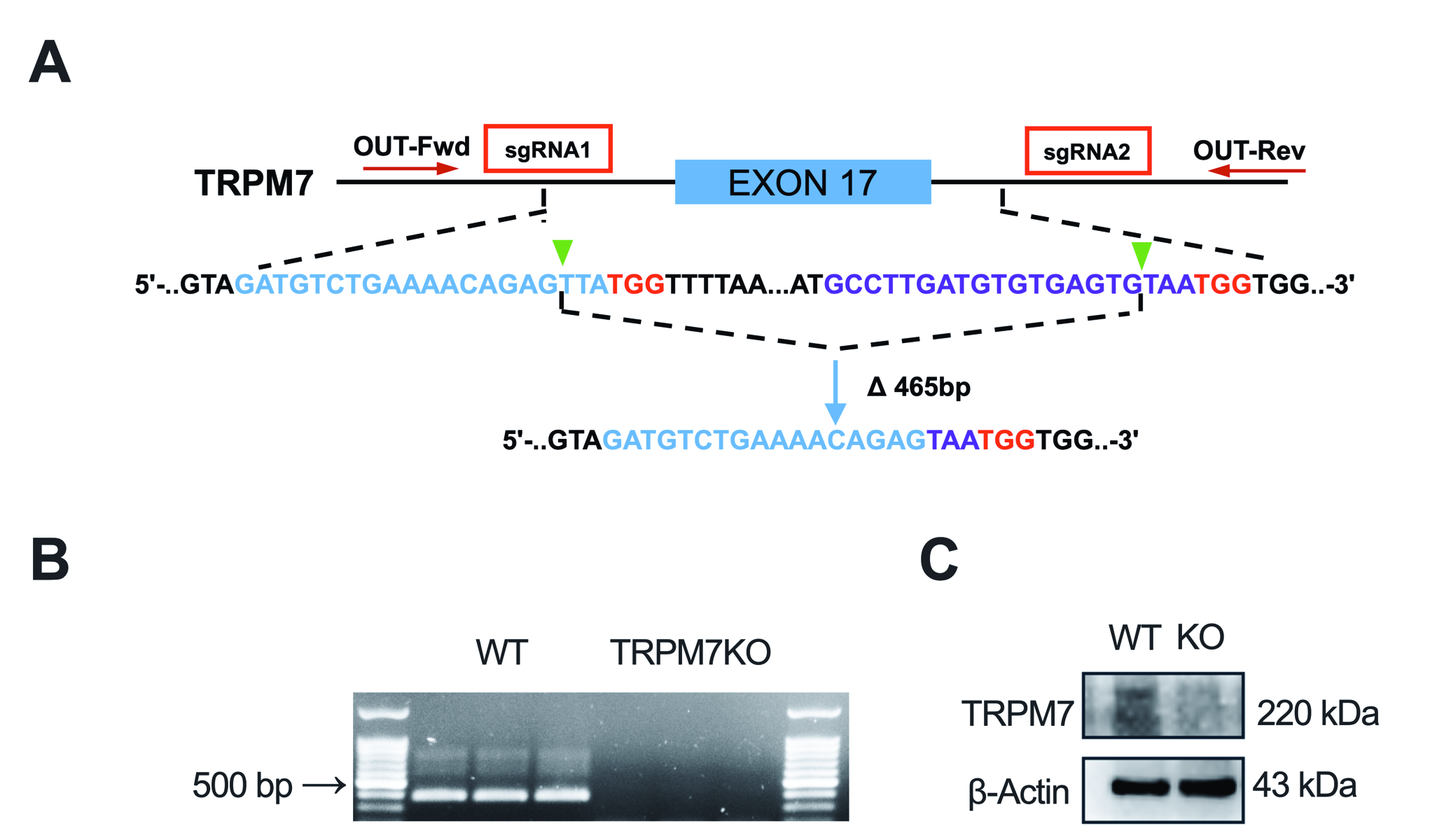


**Figure S2.** **Authentication of TRPM7 deletion in T24 cancer cell.**

**A.** Schematic showing the CRISPR/Cas9 strategy of TRPM7KO. **B.** RT-PCR of TRPM7 mRNA. **C.** Immunoblotting for TRPM7KO and WT cells.

**
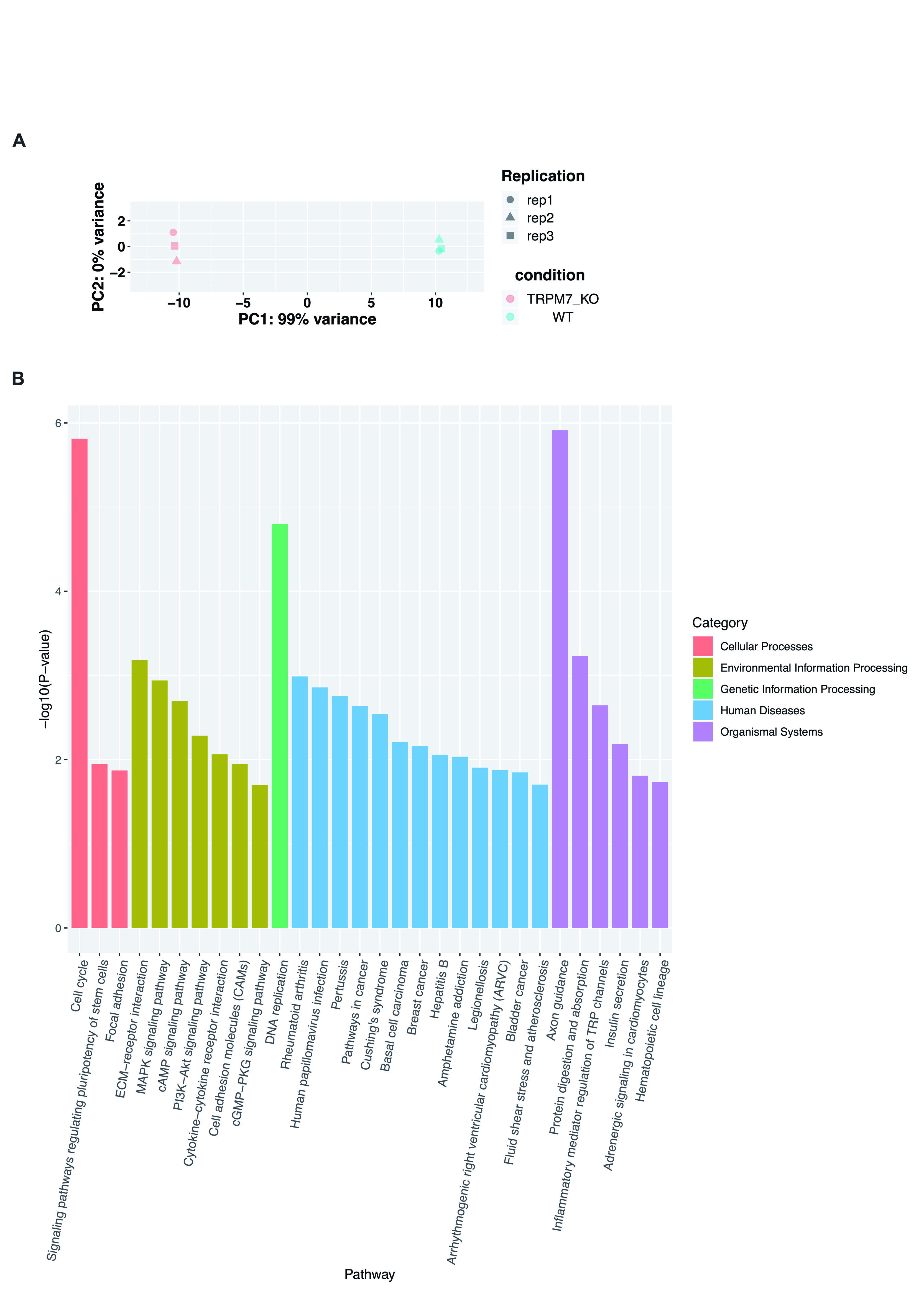
**

**Figure S3. PCA and KEGG analysis for transcriptomics.**

**A.** Principal component analysis (PCA) for RNA-seq data. **B.** Kyoto encyclopedia of genes and genomes (KEGG) analysis for RNA-seq data.

**
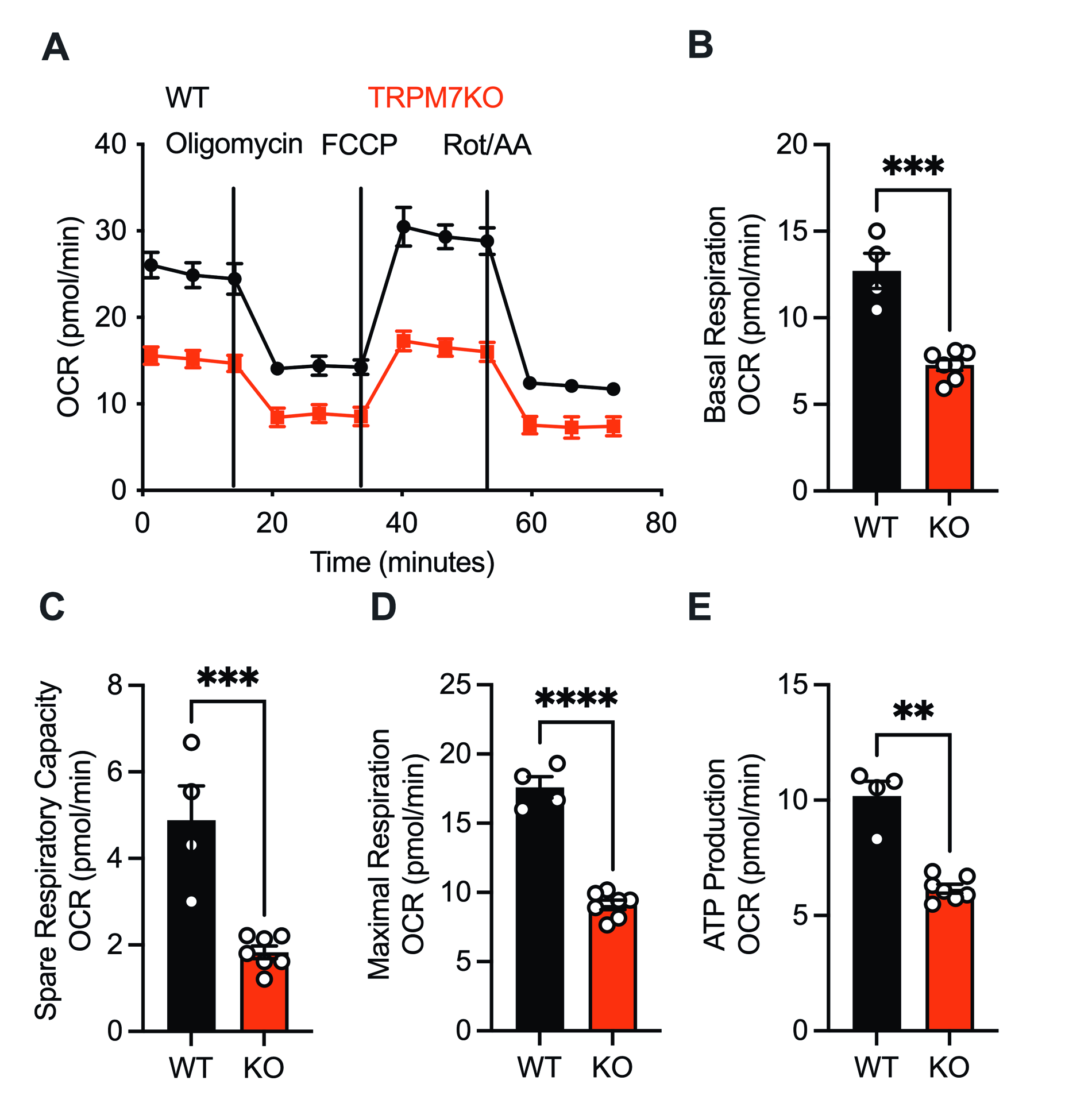
**

**Figure S4. TRPM7KO impairs glucose oxidative phosphorylation and ATP production.**

**A-E.** Lower oxidative phosphorylation and ATP production in TRPM7KO cells measured by oxygen consumption rates (OCR). Two groups of samples were compared by unpaired two-tailed Student’s *t*-test.

**
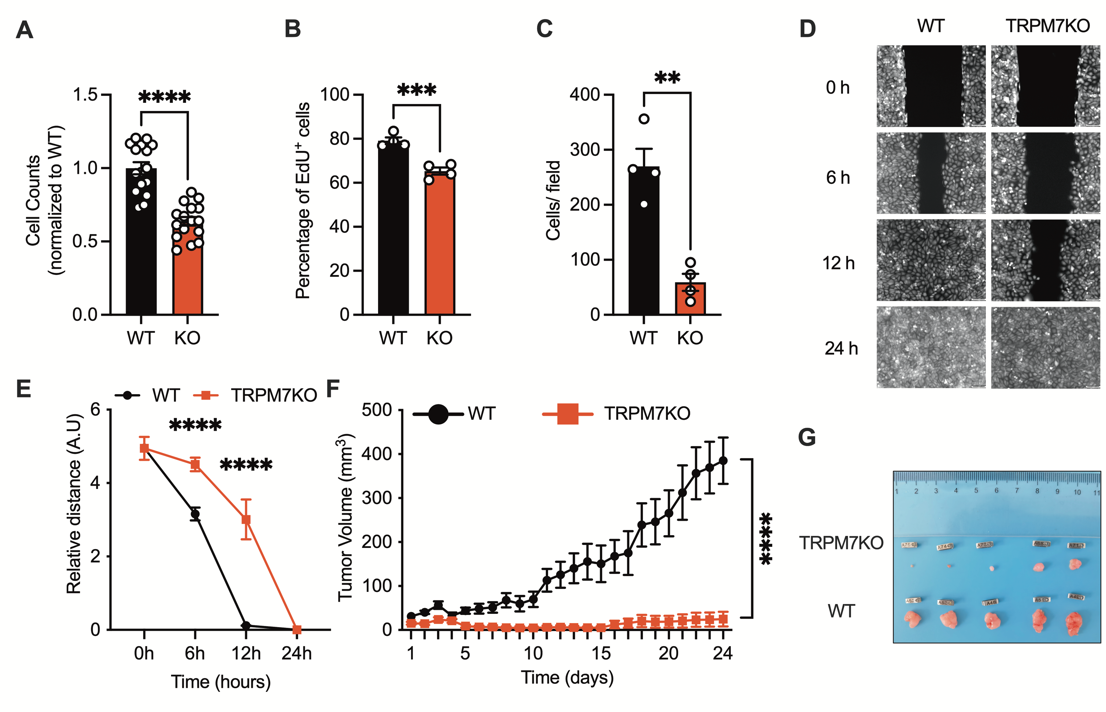
**

**Figure S5. TRPM7KO leads to tumor growth defects *in vitro* and *in vivo*.**

**A-B. Reduced** proliferation in TRPM7KO cells. **C.** Impaired invasion in TRPM7KO cells. **D-E.** Slower migration in TRPM7KO cells. **F-G.** Smaller volume of xenografted tumor derived from TRPM7KO cells (scale bars, 50 μm). Two groups of samples were compared by unpaired two-tailed Student’s *t*-test.


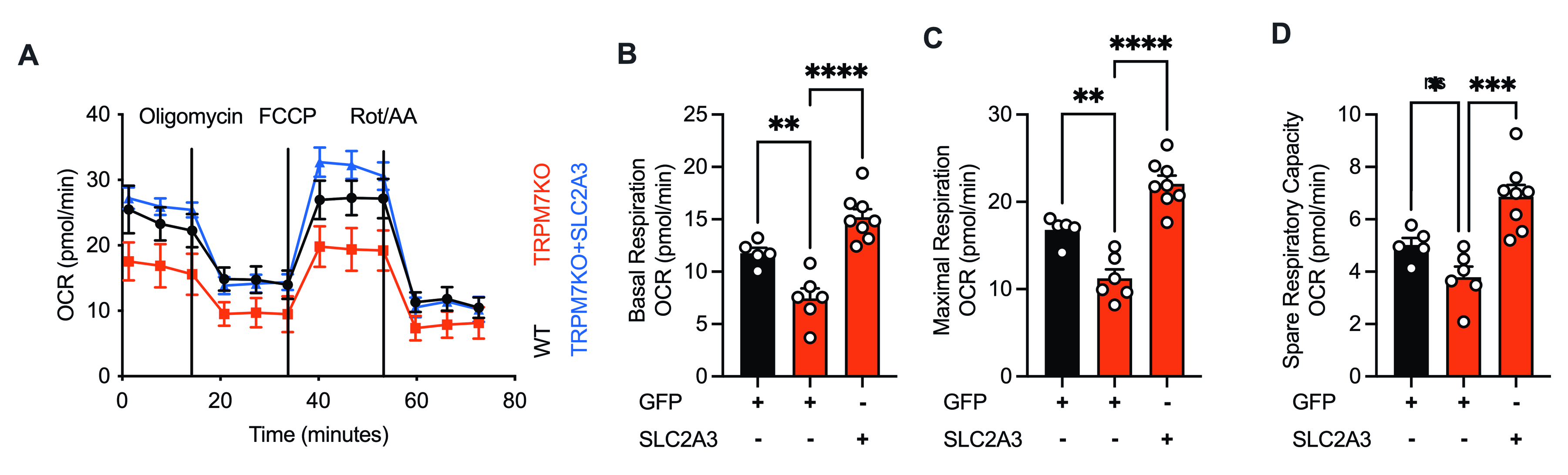


**Figure S6. SLC2A3 preserves glucose oxidative phosphorylation.**

**A-D.** Recovered glucose oxidative phosphorylation in SLC2A3-transduced TRPM7KO cells. Multiple groups were compared by ANOVA.


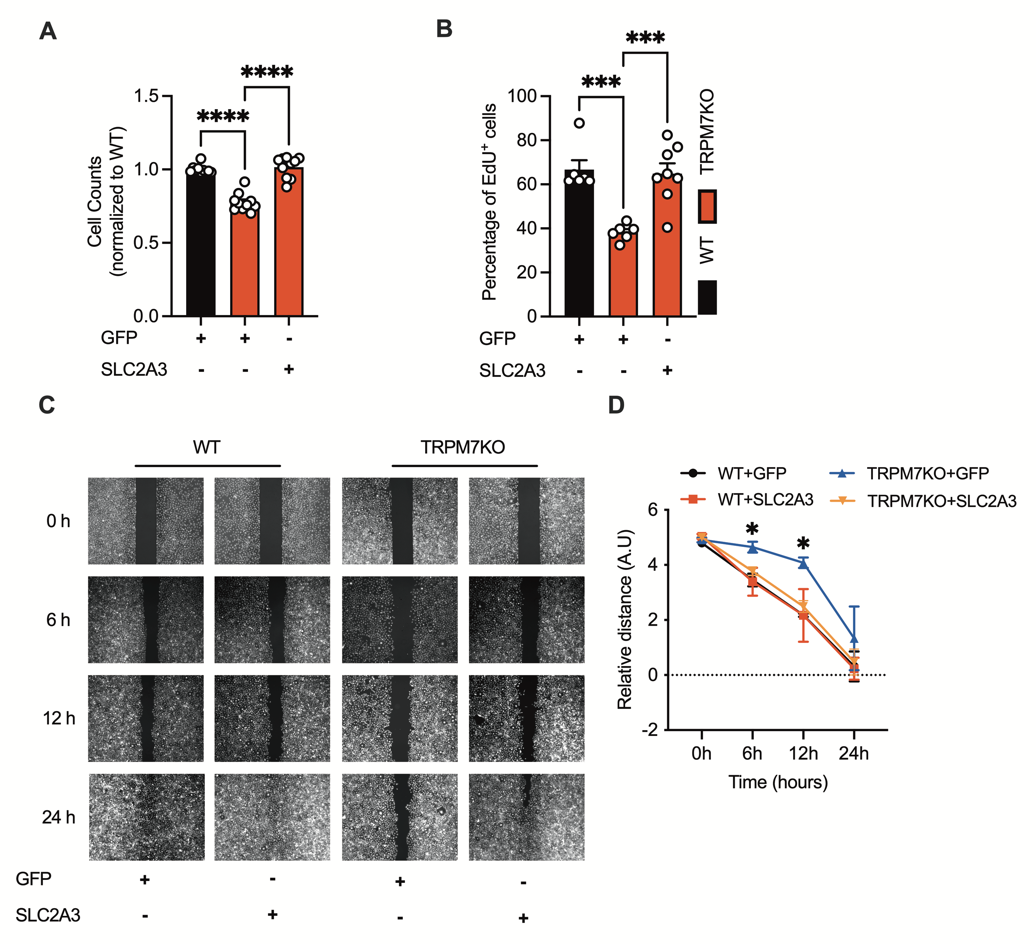


**Figure S7. SLC2A3 rescues TRPM7KO-driven tumor growth defects *in vitro*.**

**A-B.** Recovered proliferation in SLC2A3-transduced TRPM7KO cells. **C-D.** Normalized migration in SLC2A3 transduced TRPM7KO cells. Multiple groups were compared by ANOVA. **P*<0.05 showed significant difference between TRPM7KO versus TRPM7KO-transduced SLC2A3, WT respectively in Fig S7D.

**
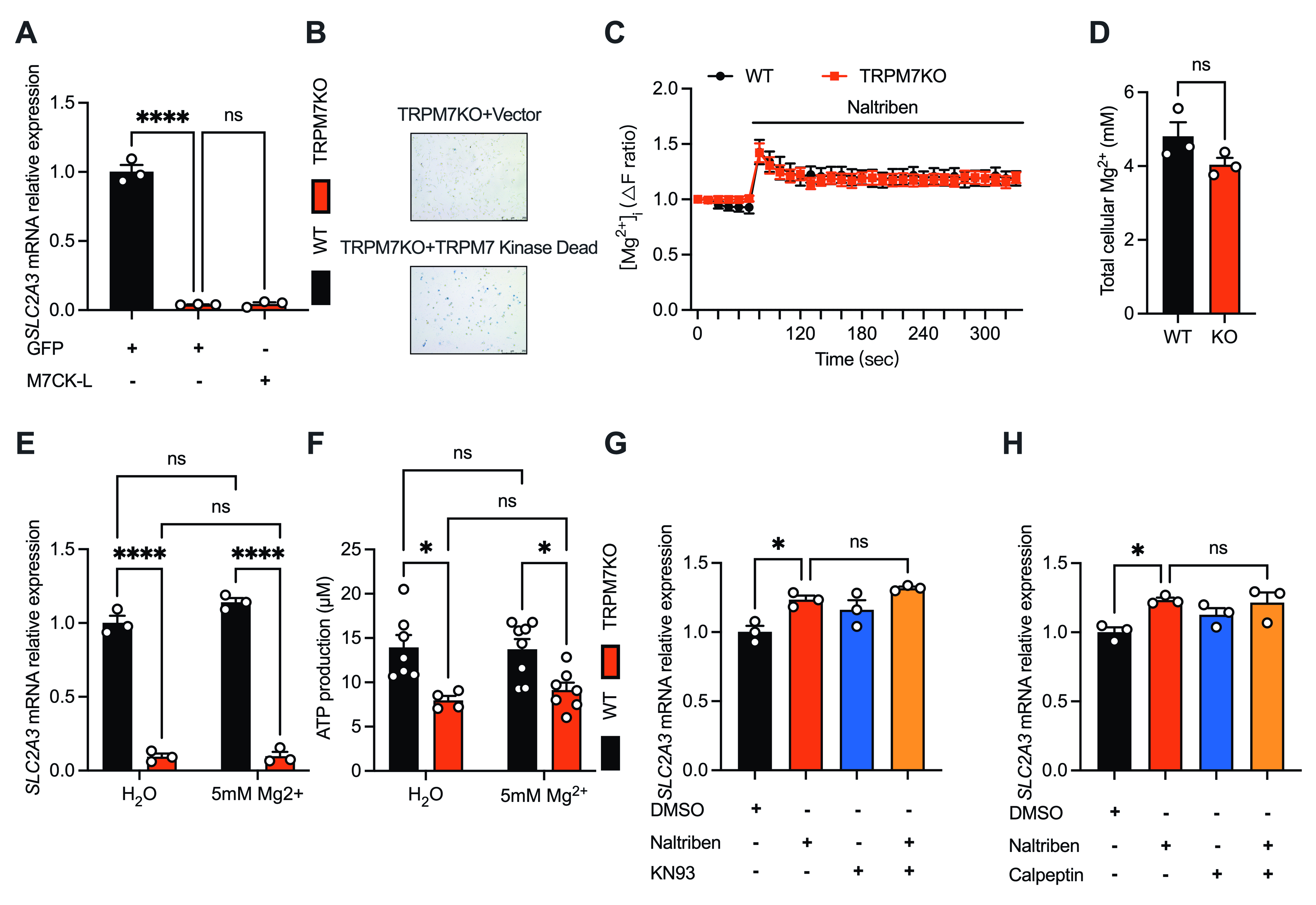
**

**Fig. S8. Mg^2+^ or the kinase function is dispensable for TRPM7-dependent regulation of SLC2A3.**

**A.** Effect of TRPM7 cleaved kinase fragment containing amino acids 1299-1864 (M7CK-L) on SLC2A3 transcription (n = 3). B. Increased cell death in TRPM7 kinase dead mutant transduced TRPM7KO cells, measured by trypan blue staining. **C.** [Mg^2+^]_i_ changes in response to naltriben (25 μM). **D.** Basal total cellular Mg^2+^ in TRPM7KO cells. **E-F.** Effect of supplemented MgSO_4_ on SLC2A3 expression and ATP production in WT and TRPM7KO cells. **G-H.** Effect of KN93 (preincubation, 10 μM, 2 h) and calpeptin (preincubation, 10 μM, 2 h) in naltriben-induced (25 μM) SLC2A3 rise. Two groups of samples were compared by unpaired two-tailed Student’s *t*-test.

Multiple groups were compared by ANOVA.


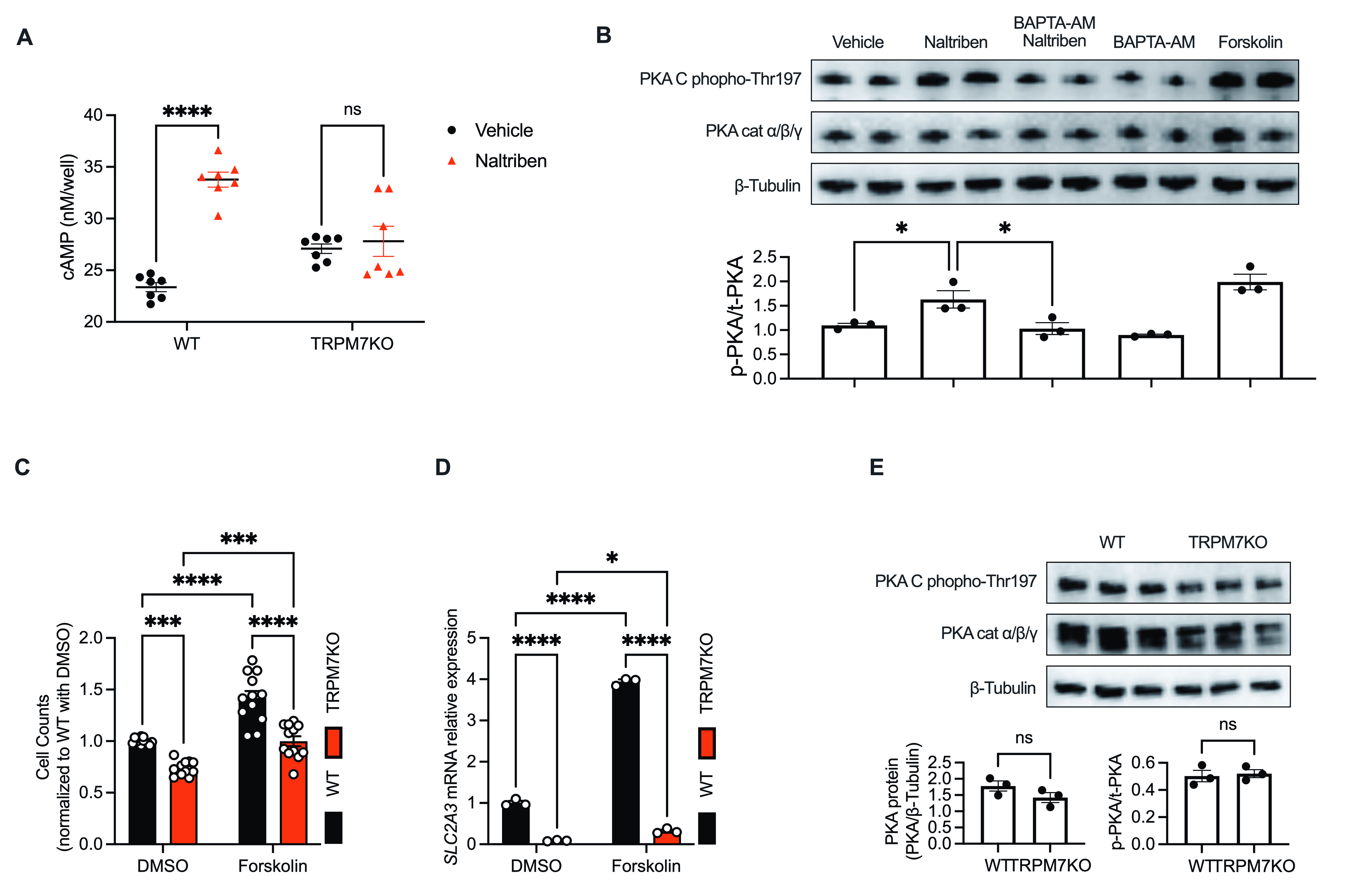


**Figure S9. The role of cAMP/PKA axis in TRPM7-mediated SLC2A3 regulation.**

**A.** Intracellular cAMP changes upon naltriben incubation. **B.** Phosphorylation of PKA under TRPM7 activation. **C.** Forskolin enhanced proliferation in TRPM7KO cells. **D.** Forskolin partially counteracted SLC2A3 downregulation in TRPM7KO cells. **E.** TRPM7KO affected neither total PKA nor phosphorylated PKA. Two groups of samples were compared by unpaired two-tailed Student’s *t*-test. Multiple groups were compared by ANOVA.


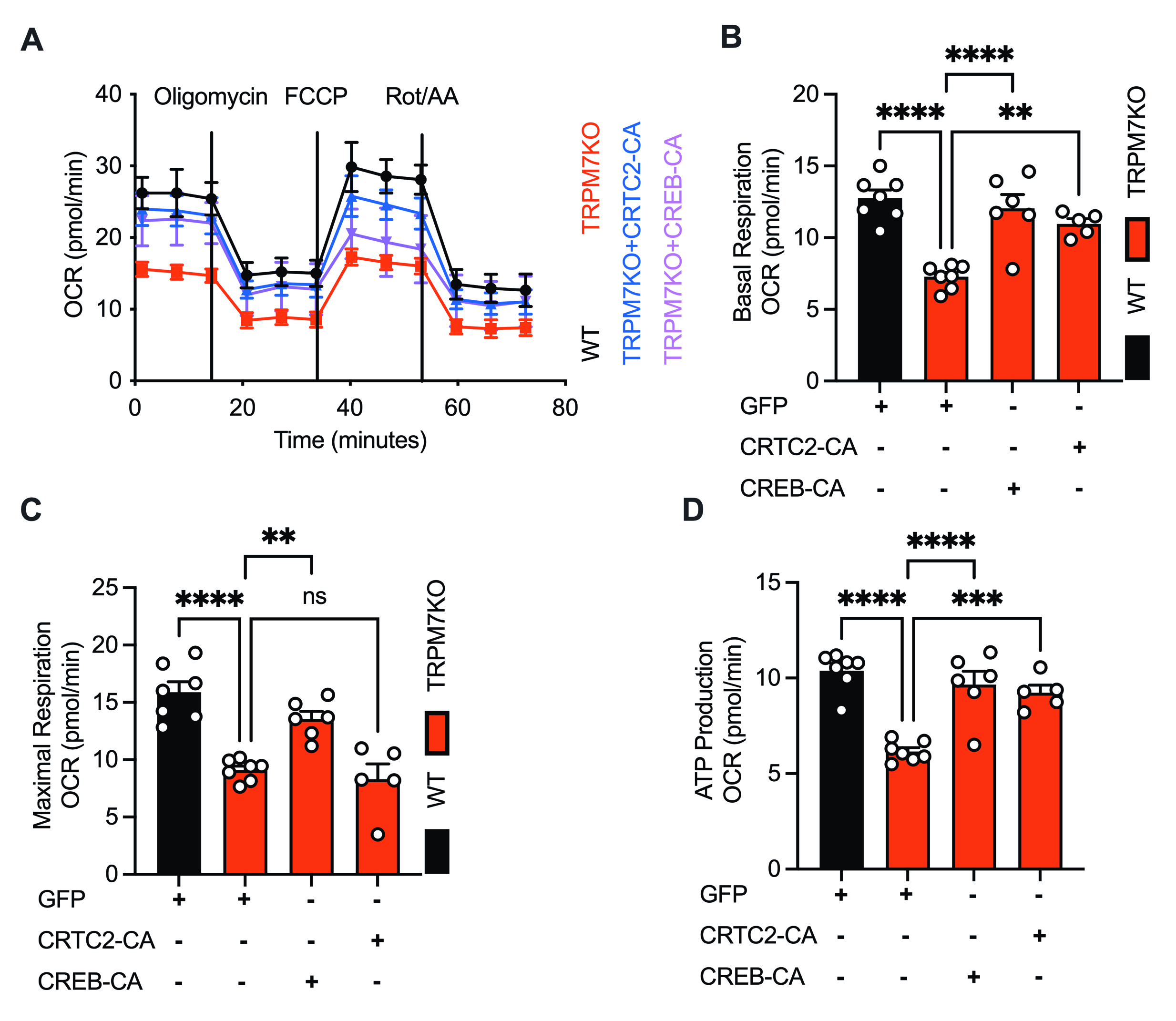


**Figure S10. CRTC2 and CREB rescue glucose oxidative phosphorylation and ATP production in TRPM7KO cells.**

**A-D.** Recovered tricarboxylic acid cycle and ATP production in CRTC2 and CREB-transducedTRPM7KO cells. Multiple groups were compared by ANOVA.


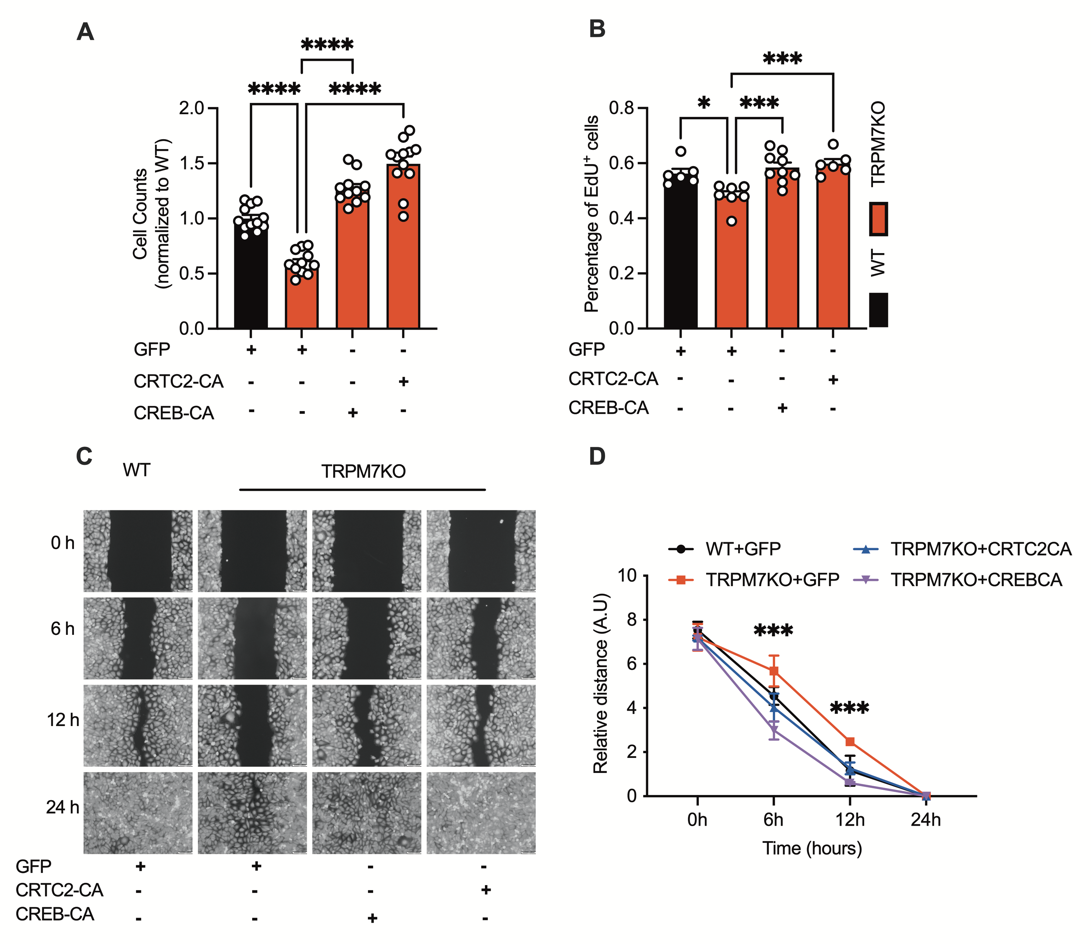


**Figure S11. Normalized tumor growth in CRTC2 and CREB-transduced TRPM7KO cells.**

**A-B.** Recovered proliferation in CRTC2 and CREB-transduced TRPM7KO cells. **C-D.** Accelerated migration in CRTC2 and CREB-transduced TRPM7KO cells. ****P*<0.001 denotes a significant statistical difference between TRPM7KO versus CRTC2 or CREB -transduced TRPM7KO cells. Multiple groups were compared by ANOVA.


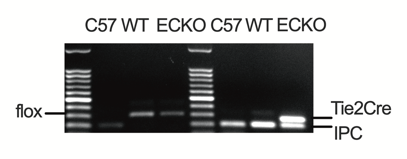


**Figure S12.** Genotyping for TRPM7 endothelial deletion mice. IPC, internal positive control.


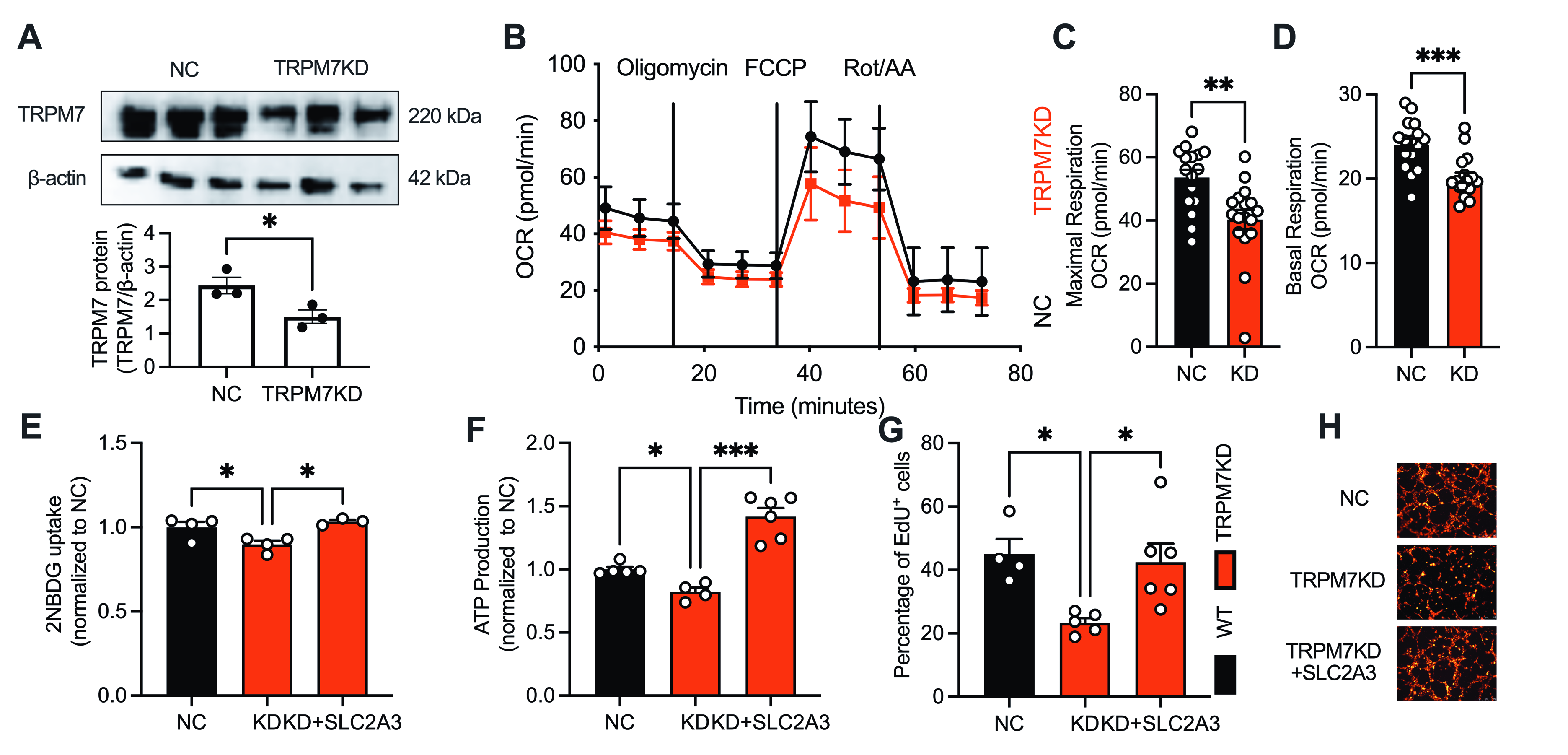


**Figure S13. TRPM7 regulates cell growth and endothelial glycolysis via SLC2A3 in HUVECs.**

**A.** Blotting of TRPM7KD efficiency. **B-D.** Lower oxidative phosphorylation in TRPM7 siRNA-transfected HUVECs. **E-H.** Normalized cell cycle, tube formation (scale bars, 100 μm), glucose uptake and ATP production in lenti-hSLC2A3-transduced TRPM7 knockdown HUVECs. Two groups of samples were compared by unpaired two-tailed Student’s *t*-test. Multiple groups were compared by ANOVA.


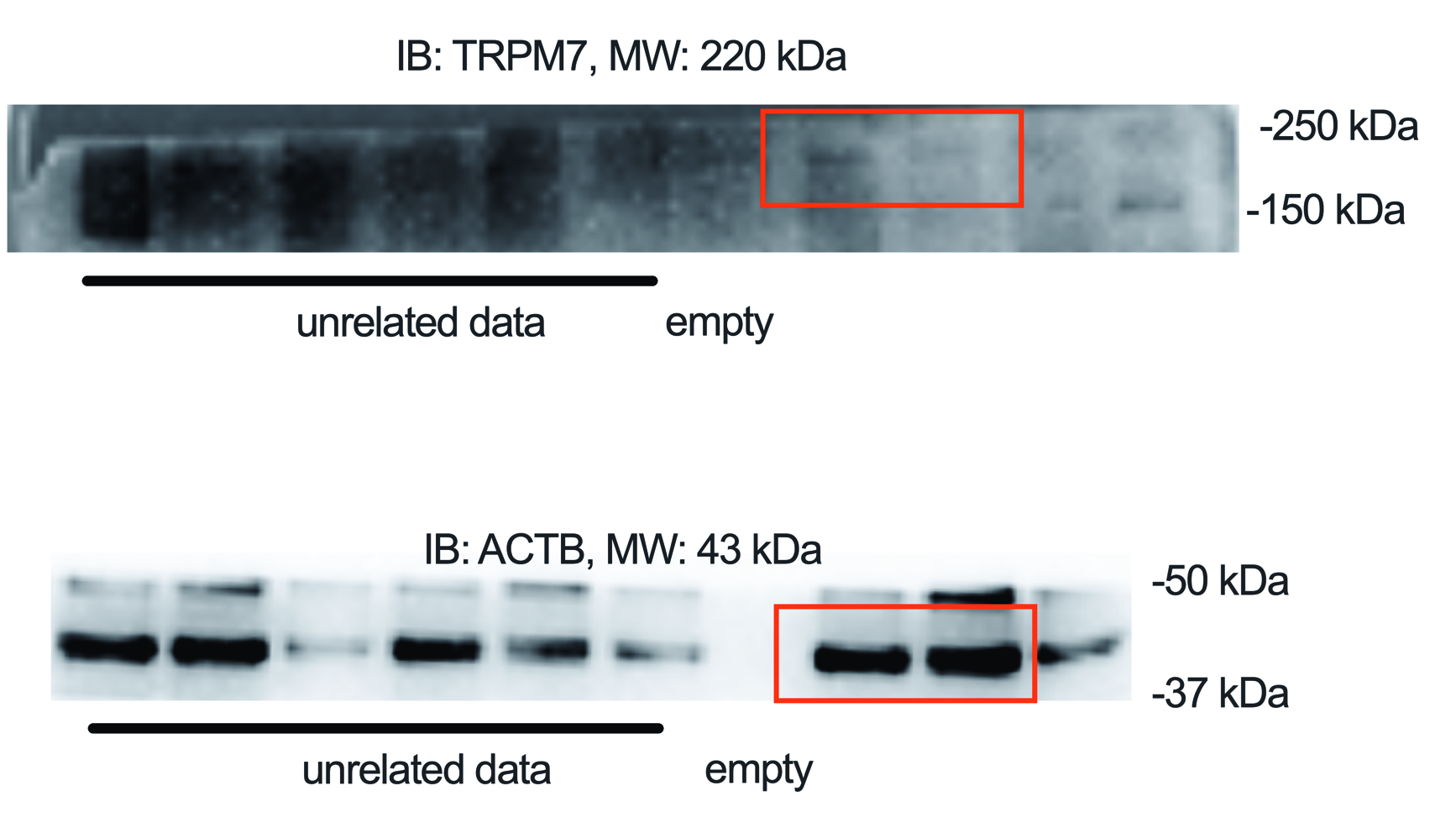


**Figure S14. Whole blotting of Figure S2C.**

**
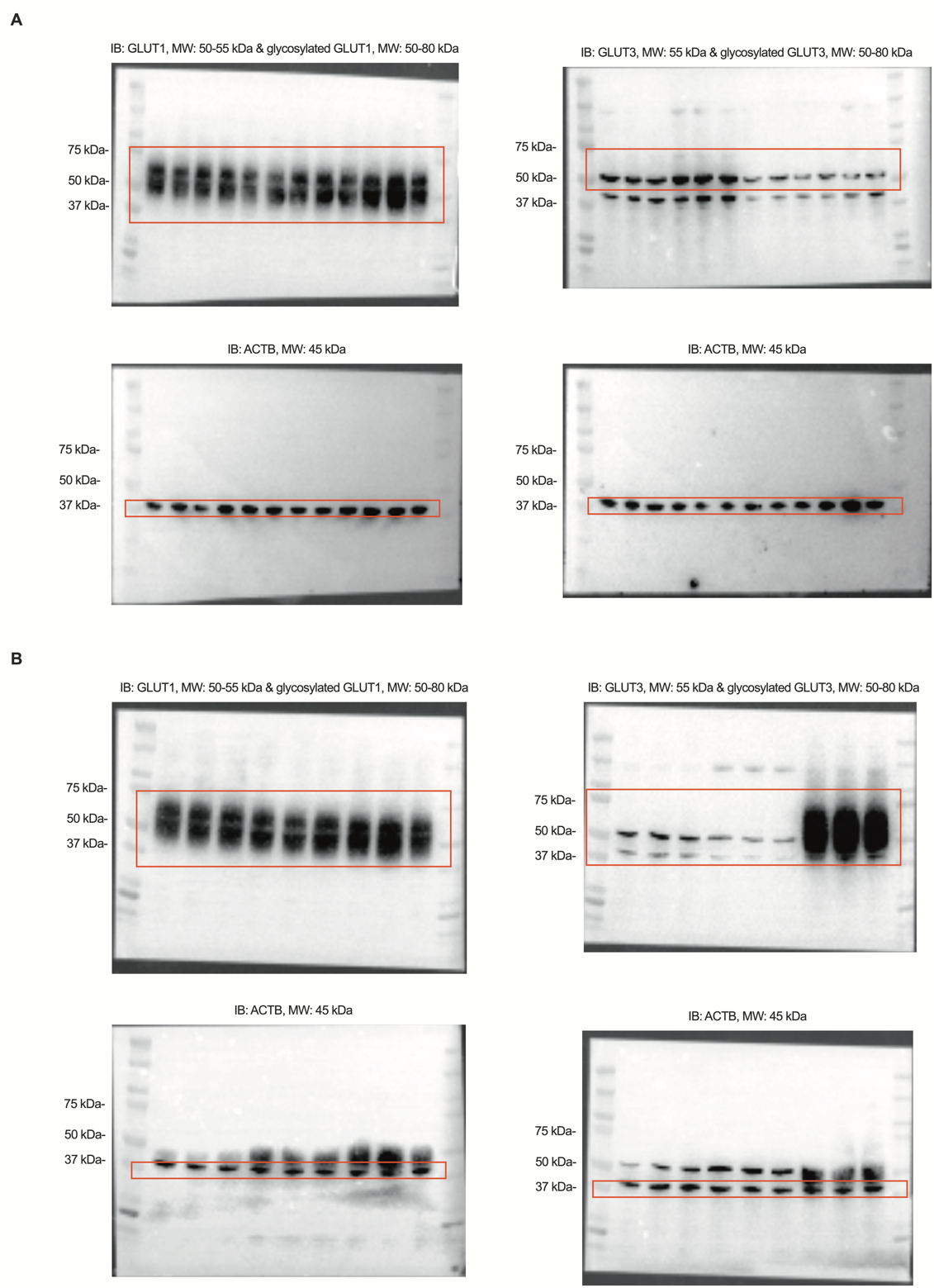
Figure S15. Raw data of WB.**

**A.** Whole blotting of Figure 2E. **B.** Whole blotting of Figure 3A.

**
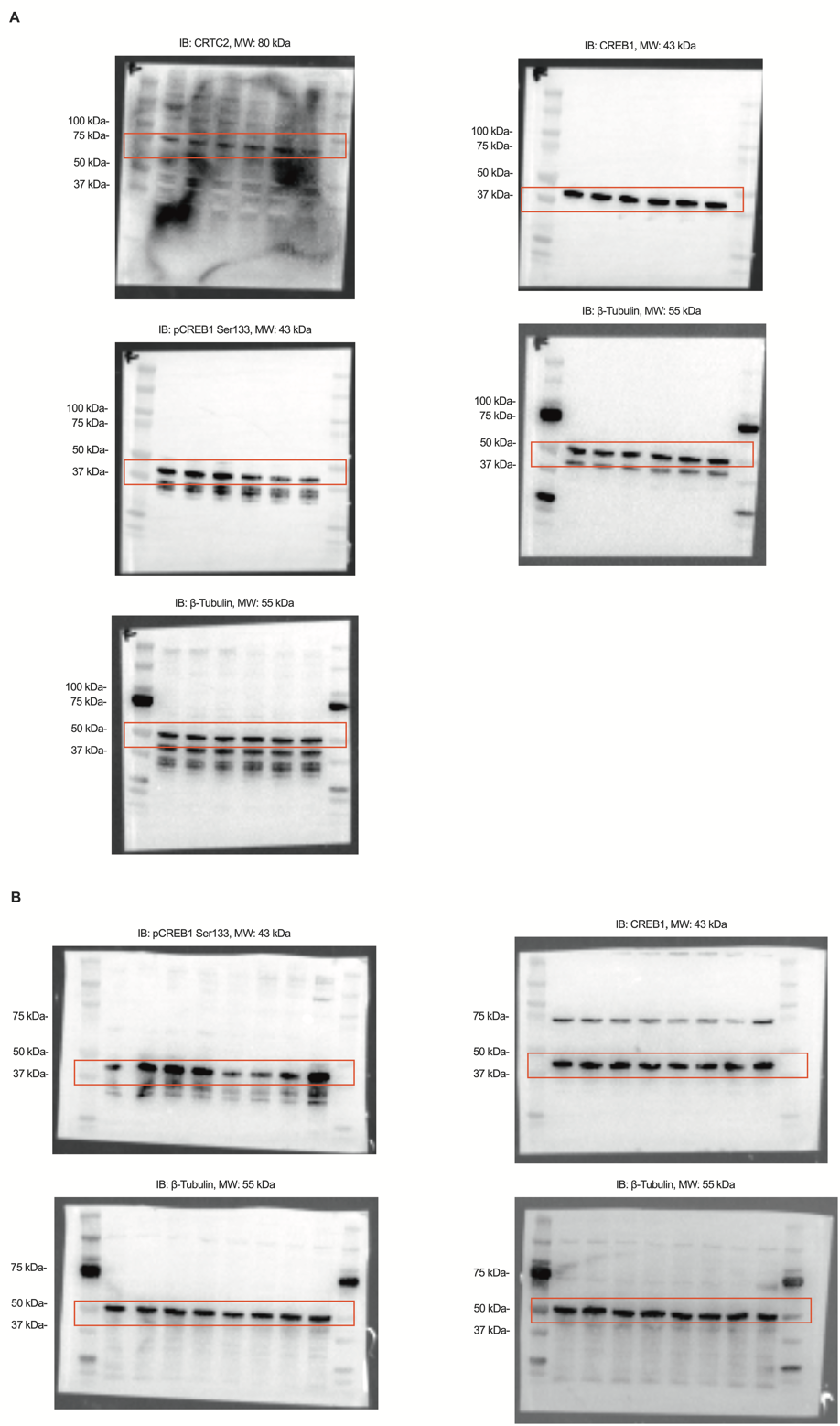
**

**Figure S16. Raw data of WB.**

**A.** Whole blotting of Figure 5B. **B.** Whole blotting of Figure 5D.

**
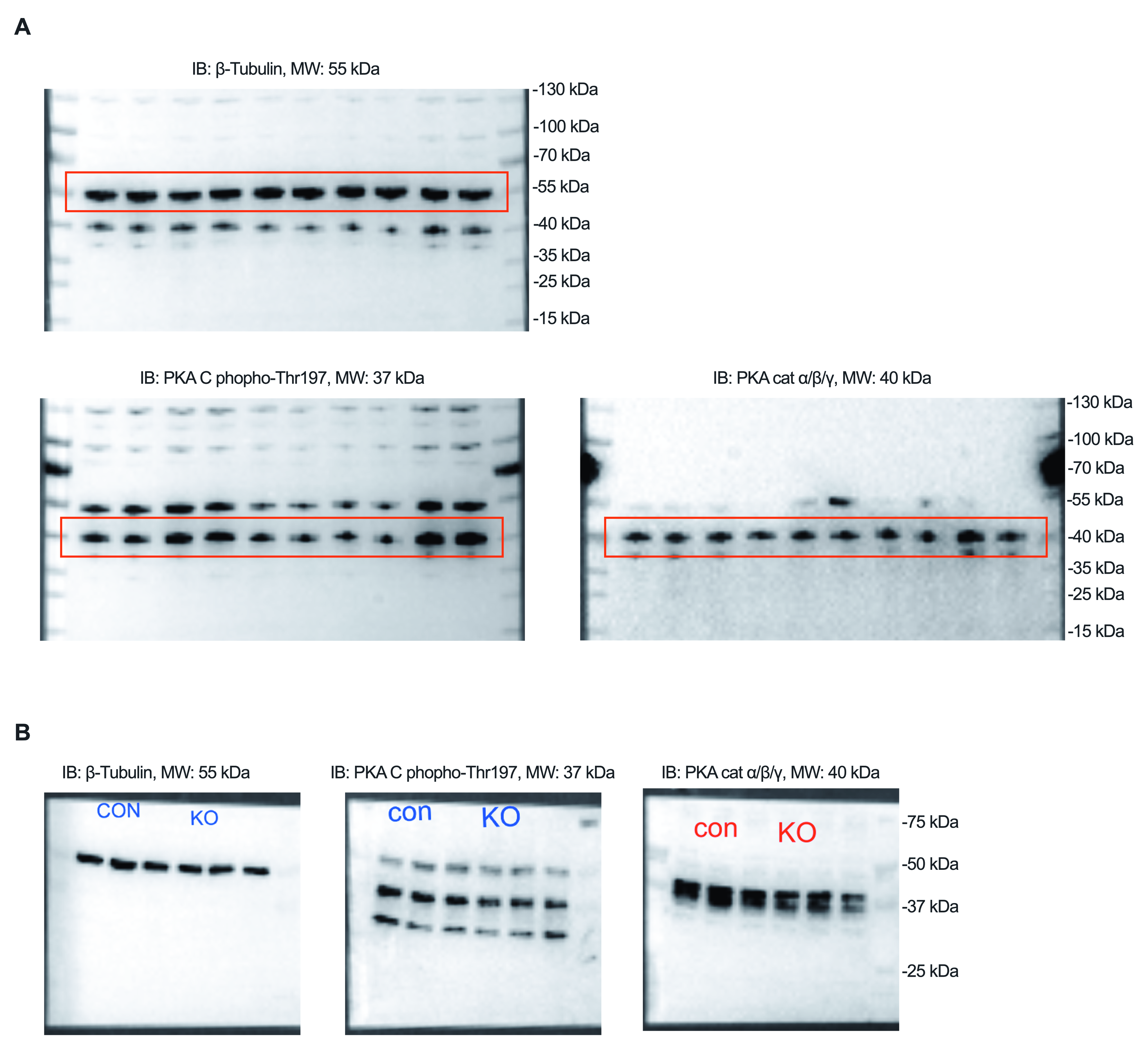
**

**Figure S17. Raw data of WB.**

**A.** Whole blotting of S9B. **B.** Whole blotting of S9E.


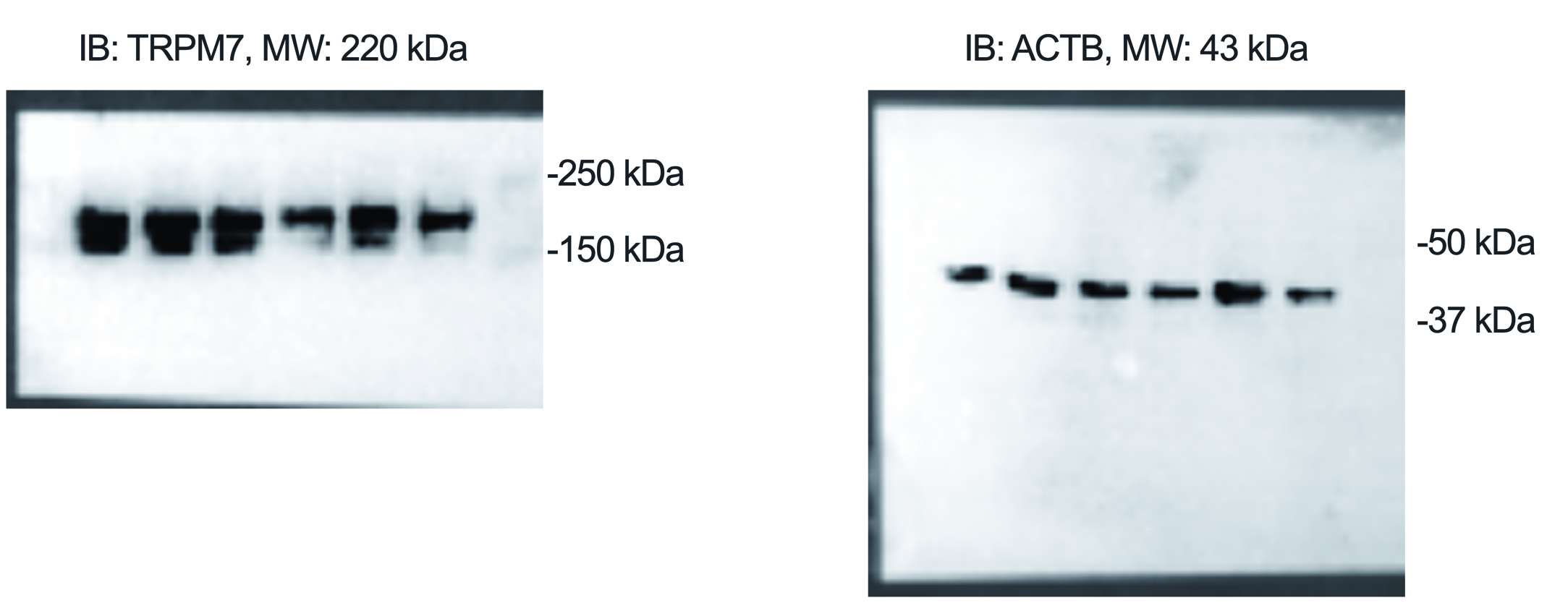


**Figure S18. Raw data of WB.** Whole blotting of S13A.
